# Chemoenzymatic Synthesis of Enantiomeric, Bicyclic δ-Halo-γ-lactones with a Cyclohexane Ring, Their Biological Activity and Interaction with Biological Membranes

**DOI:** 10.3390/biom10010095

**Published:** 2020-01-06

**Authors:** Marcelina Mazur, Aleksandra Włoch, Fouad Bahri, Hanna Pruchnik, Aleksandra Pawlak, Bożena Obmińska-Mrukowicz, Gabriela Maciejewska, Witold Gładkowski

**Affiliations:** 1 Department of Chemistry, Wrocław University of Environmental and Life Sciences, Norwida 25, 50-375 Wrocław, Poland; witold.gladkowski@upwr.edu.pl; 2 Department of Physics and Biophysics, Wrocław University of Environmental and Life Sciences, Norwida 25, 50-375 Wrocław, Poland; aleksandra.wloch@upwr.edu.pl (A.W.); hanna.pruchnik@upwr.edu.pl (H.P.); 3 Laboratory of Microbiology and Plant Biology, Faculty of Natural and Life Sciences, University of Abd El Hamid Ibn Badiss of Mostaganem, Mostaganem 27000, Algeria; bahrifouad13@gmail.com; 4 Department of Pharmacology and Toxicology, Wrocław University of Environmental and Life Sciences, Norwida 31, 50-375 Wrocław, Poland; aleksandra.pawlak@upwr.edu.pl (A.P.); bozena.obminska-mrukowicz@upwr.edu.pl (B.O.-M.); 5 Central Laboratory of the Instrumental Analysis, Wrocław University of Science and Technology, Wybrzeże Wyspiańskiego 27, 50-370 Wrocław, Poland; gabriela.maciejewska@pwr.edu.pl

**Keywords:** lactones, halolactonisation, kinetic resolution ofalcohols, antimicrobial activity, antiproliferative activity, hemolytic activity, erythrocytes, biological membranes

## Abstract

Starting from 1-acetyl-1-cyclohexene, three enantiomeric pairs (ee ≥ 99%) of bicyclic δ-halo-γ-lactones with cyclohexane ring were obtained in five-step synthesis. The key stereochemical steps were lipase-catalyzed kinetic resolution of racemic 1-(cyclohex-1-en-1-yl) ethanol followed by transfer of chirality to ethyl 2-(2-ethylidenecyclohexyl) acetate in the Johnson–Claisen rearrangement. Synthesized halolactones exhibited antiproliferative activity towards canine B-cell leukemia cells (GL-1) and canine B-cell chronic leukemia cells (CLB70) and the most potent (IC_50_ 18.43 ± 1.46 μg/mL against GL-1, IC_50_ 11.40 ± 0.40 μg/mL against CLB70) comparable with the control etoposide, was (1*R*,6*R*,1′*S*)-1-(1′-chloroethyl)-9-oxabicyclo[4.3.0]nonan-8-one (**8b**). All halolactones did not have a toxic effect on erythrocytes and did not change the fluidity of membranes in the hydrophobic region of the lipid bilayer. Only weak changes in the hydrophilic area were observed, like the degree of lipid packing and associated hydration. The racemic halolactones were also tested for their antimicrobial properties and found to exhibit selectivity towards bacteria, in particular, towards *Proteus mirabilis* ATCC 35659.

## 1. Introduction

Lactones are cyclic esters which are detected in plants and animals. The natural lactones isolated from fruits and essential oils can be used as fragrance components, like jasmine lactone or as a flavor in the food industry (δ-decalactone) [1,2,3]. Those compounds can also be pheromones [4,5] or antifeedants [6,7,8] but the largest interest is put in their antimicrobial and cytotoxic potential. There are numerous examples presenting the antibacterial and antifungal activity of lactones [8,9]. Some phthalides derivatives are active against phytopathogenic fungal strains from the genus *Fusarium* [10]. Vernolide and vernodalin, identified in the extract from *Vernonia colorata*, exhibit antibacterial activity against Gram-positive bacteria *Staphylococcus aureus* and *Bacillus subtilis* [11,12]. The natural occurring lactones are proved to be cytotoxic against numerous cancer cell lines. In this group, the most potent are the sesquiterpene lactones like costunolide, parthenolide, helenalin or artemisinin [13,14,15].

On the other hand, many efforts are currently being made to obtain synthetic lactones with simplified structure and high cytotoxic activity. This approach is reasonable in the context of low concentrations of natural active substances in plant material and high cost and difficulties during the isolation processes. In the last few years, our scientific interests have concentrated on the synthesis of biologically active lactones [7,8,16]. In our previous research on antiproliferative compounds, we developed a synthetic pathway to obtain highly active γ-lactones with a β-aryl substituent [17,18,19]. An important element of the structure of these compounds, affecting their activity, was the presence of a halogen atom. Recently, we have also reported that some bicyclic γ-lactones with a cyclohexane system exhibited cytotoxicity against selected cancer cell lines [20]. Assuming that the introduction of a halogen atom can potentially increase the cytotoxic effect exhibited by this group of compounds, we designed the synthesis in order to obtain a series of new halogen derivatives of γ-lactones with a cyclohexane ring (**6**–**8**), containing iodine, bromine and chlorine atoms in the lactone structure. The next important aspect was a comparison of biological activity of opposite enantiomers. Application of lipase-catalyzed kinetic resolution of their precursor, racemic allylic alcohol, allowed us to prepare pairs of lactone enantiomers and consequently check the significance of configuration of stereogenic centers.

The cytotoxic activity of chemotherapeutic agents is related to different mechanisms which lead to programmed cell death. As the biological membrane is an important barrier, which protects the internal cell structures and allows them to function properly, understanding its interaction with cytotoxic agents is extremely important. In particular, the red blood cells (RBC) and membranes of RBC appear to be an excellent model to evaluate toxicity of molecules by measuring cell damage and analyzing membrane biophysical parameters.

The main goal of this research was the design and synthesis of new optically active δ-halo-γ-lactones with a cyclohexane ring and the evaluation of their biological activity, namely, antimicrobial, antiproliferative and hemolytic. The second important aspect was the attempt to explain the mechanism of interactions of the tested compounds with biological membranes. Therefore, the effects of the synthesized lactones on erythrocyte membrane fluidity, hydration and packing arrangement of lipids were investigated.

## 2. Materials and Methods

### 2.1. Analysis

The reactions’ progress and the purity of obtained products were analyzed by TLC (DC-Alufolien Kieselgel 60 F254, Merck, Darmstadt, Germany. GC analysis was registered on an Agilent Technologies 6890N instrument (Santa Clara, CA, USA) with FID detector, the hydrogen was used as a carrier gas. Purity of obtained compounds as well as progress of the reactions were checked on DB-5HT column (Agilent, Santa Clara, CA, USA, polyimide-coated fused silica tubing, 30 m × 0.25 mm × 0.10 × µm). The analytical parameters: detector 300 °C, injector 250 °C, temperature programme: 80 °C (0 min), 80–200 °C (25 °C/min), 200–300 °C (30 °C/min), 300 °C (2 min).

The enantiomeric excesses were calculated based on chiral GC analysis applying CP Chirasil-Dex CB column (25 m × 0.25 mm × 0.25 µm). The analytical conditions were as follows: injector 200 °C, detector (FID) 250 °C, temperature programme: 75 °C (1 min), 75–140 °C (0.3 °C/min), 140–200 (5 °C/min), 200 °C (10 min).

The products were purified using column chromatography on silica gel (Kieselgel 60, 230–400 mesh, Merck).

Nuclear magnetic resonance spectra (^1^H, ^13^C-NMR, COSY, HMBC, and HMQC) were registered on a Bruker Avance II 600 MHz spectrometer (Bruker, Rheinstetten, Germany) in CDCl_3_ solutions. As references, signals of residual solvent (δ_H_ = 7.26, δ_C_ = 77.0) were used. High-resolution mass spectra (HRMS) were registered on spectrometer Waters ESI-Q-TOF Premier XE (Waters Corp., Millford, MA, USA) using electron spray ionization (ESI) technique.

IR spectra were recorded using Mattson IR 300 Thermo Nicolet spectrophotometer (Thermo Scientific^™^, Waltham, MA, USA). The melting points (uncorrected) were measured on a Boetius apparatus.

### 2.2. Chemicals

1-Acetyl-1-cyclohexene (≥98% purity), propionic acid (≥99.5% purity), *N*-bromosuccinimide (NBS), *N-C*hlorosuccinimide (NCS) were purchased from Sigma–Aldrich. Triethylorthoacetate (≥98% purity) were purchased from Fluka. Iodine, potassium iodide, hydrochloric acid, sodium bicarbonate, sodium hydroxide and sodium thiosulphate were purchased from POCh (Gliwice, Poland). All the solvents used in column chromatography, purchased from Chempur, were of analytical grade.

### 2.3. Synthesis of Lactones with Cyclohexane System

Three pairs of chiral bicyclic lactones with cyclohexane system were prepared in six steps chemoenzymatic synthesis.

#### 2.3.1. 1-(Cyclohex-1-en-1-yl) ethanol (**2**)

Known unsaturated alcohol (**2**) was obtained as a product of 1-acetyl-1-cyclohexene reduction (isolated yield 90%) with sodium borohydride, according to procedure describe earlier by Mazur et al. [7]. The spectroscopic data of *rac*-**2** correspond to literature data [21,22].

#### 2.3.2. Kinetic Resolution of rac-2 by Enzymatic Transesterification

The substrate for the lipase catalysed kinetic resolution was racemic alcohol (**2**) (5.5 g, 40 mmol). The reaction was performed in diisopropyl ether (50 mL) using lipase CAL-B from *Candida antarctica* (10 wt.-% of alcohol) and vinyl propionate (6 mL, 56 mmol) as the acyl donor. After 2 h at room temperature the reaction was finished. The lipase was separated by filtration and the organic solvents were evaporated in vacuo. The mixture of (*S*)-alkohol (**2a**) and (*R*)-propionate (**3**) was separated by column chromatography (hexane:acetone 20:1). The spectroscopic data of alcohol **2a** and propionate **3** are in accordance with literature reports [21,23].

(−)-(*S*)-1-(cyclohex-1-en-1-yl)ethanol (**2a**): $\alpha_{D}^{25}$ = −8.9 (c = 0.55, CHCl_3_, ee = 98.1%), [ref. [21] $\alpha_{D}^{25}$ = −9.4 (c = 1.5, CHCl_3_, ee = 91%)];

(+)-(*R*)-1-(cyclohex-1-en-1-yl)ethyl propionate (**3**): $\alpha_{D}^{25}$ = +58.5 (c = 0.65, CHCl_3_, ee = 99%);

#### 2.3.3. (R)-1-(cyclohex-1-en-1-yl)ethanol (**2b**)

The ester **3** (3 g, 16 mmol) was refluxed in a mixture of 5% ethanolic solution of NaOH (60 mL) and water (10 mL). When the reaction was finished (3 h, TLC), the solvent was evaporated and the residue was resuspended with water. The alcohol **2b** was extracted with dichloromethane (3 × 40 mL) and the organic phase were washed with brine and dried over anhydrous MgSO_4_. After solvent evaporation (*R*)-alcohol **2b** was obtained (1.7 g, isolated yield 84%).

(*R*)-1-(cyclohex-1-en-1-yl)ethanol (**2b**): $\alpha_{D}^{25}$ = +9.7 (c = 1.25, CHCl_3_, ee = 98.7%), [ref. [23] $\alpha_{D}^{25}$ = +6.4 (c = 1.2, CHCl_3_, ee = 68%)];

#### 2.3.4. Ethyl 2-(2-ethylidenecyclohexyl) Acetates (**4a** and **4b**)

##### General Procedure

A solution of alcohol **2** (17.5 mmol) in triethylorthoacetate (15 mL) and drop of propionic acid was heated in 138 °C with continuous distilling off the ethanol. After 24 h the crude product was purified by column chromatography (silica gel, hexane:acetone 10:1) and the pure ester **4** was obtained.

(−)-(1*S*,2*E*)-Ethyl 2-(2-ethylidenecyclohexyl) acetate (**4a**), Obtained from alcohol **2a** (2.2 g, 17.5 mmol), yield 2.8 g (82%); Rt = 84 min, $\alpha_{D}^{25}$ = −13 (c = 0.75, CHCl_3_, ee = 98%); HRMS: (ESI-TOF) *m*/*z* [M + Na]^+^ calcd for C_12_H_20_O_2_Na 219.1361; found 219.1355, other spectroscopic data consistent with literature reports [24,25].

(+)-(1*R*,2*E*)-Ethyl 2-(2-ethylidenecyclohexyl)acetate (**4b**), Obtained from alcohol **2b** (1.7 g, 13.6 mmol), yield 2.1 g (87%); Rt = 81 min, $\alpha_{D}^{25}$ = +14 (c = 0.8, CHCl_3_, ee = 99%); spectroscopic data identical to the values reported for (−)-(1*S*,2*E*)-**4a**

#### 2.3.5. (E)-2-(2-Ethylidenecyclohexyl) Acetic Acid (**5a** and **5b**)

##### General Procedure

The ester **4** was refluxed in 2.5% a ethanolic solution of KOH (50 mL) for 5 h. When the reaction was finished, solvent was evaporated, the residue was resuspended in water and then extracted three times with diethyl ether (Et_2_O). The aqueous layer was acidified (1 M HCl) and the carboxylic acid was extracted three times with Et_2_O. The combined organic fractions were washed with NaCl and dried over anhydrous magnesium sulphate. After evaporating the solvent, a pure acid was obtained.

(−)-(*S*, *E*)-2-(2-Ethylidenecyclohexyl) acetic acid (**5a**), Obtained from ester **4a** (2.8 g, 14.4 mmol), yield 2.2 g (94%); Rt = 186 min, $\alpha_{D}^{25}$ = −18 (c = 0.45, CHCl_3_, ee > 99%); HRMS: (ESI-TOF) *m*/*z* [M − H]^−^ calcd for C_10_H_16_O_2_ 241.1046; found 241.1050. The spectroscopic data are in accordance with literature reports [26].

(+)-(*R*, *E*)-2-(2-Ethylidenecyclohexyl) acetic acid (**5b**), Obtained from ester **4b** (2.1 g, 10.6 mmol), yield 1.3 g (75%); Rt = 184 min, $\alpha_{D}^{25}$ = +18 (c = 0.45, CHCl_3_, ee > 99%); spectroscopic data identical to the values reported for (−)-(*S*, *E*)-**5a**

#### 2.3.6. 1-(1′-Iodoethyl)-9-Oxabicyclo[4.3.0]nonan-8-one (**6a** and **6b**)

##### General Procedure

The mixture of acid **5**, 25 mL in Et_2_O with addition of saturated sodium bicarbonate (25 mL) was stirred at room temperature for 1 h. Then, a mixture of iodine (1.4 g) and potassium iodide (2.8 g) in H_2_O was added dropwise to the stirred solution until a stable brown color was maintained. When the reaction was completed, the mixture was diluted with Et_2_O and washed with Na_2_S_2_O_3_. The organic layer was washed with saturated sodium bicarbonate, brine and dried over MgSO_4_. The crude product was purified on silica gel (hexane:acetone, 20:1). New 1-(1-iodoethyl)-9-oxabicyclo[4.3.0]nonan-8-one was obtained as the product. The physical and spectral data of the synthesized iodolactone **6** are given below.

(−)-(1*S*,6*S*,1′*R*)-1-(1′-Iodoethyl)-9-oxabicyclo[4.3.0]nonan-8-one (**6a**), Obtained from acid **5a** (0.5 g, 3 mmol), yield 0.6 g (72%); Rt = 189 min, $\alpha_{D}^{25}$ = −54 (c = 0.45, CHCl_3_, ee > 99%); mp 104–107 °C; ^1^H-NMR (600 MHz, CDCl_3_) δ: 1.26 (m, 1H, one of CH_2_-5), 1.35 (m, 1H, one of CH_2_-4), 1.46 (m, 1H, one of CH_2_-3), 1.57–1.67 (m, 2H, one of CH_2_-3 and one of CH_2_-4), 1.81 (m, 1H, one of CH_2_-5), 1.94 (d, 3H, *J* = 7.0, CH_3_-2′), 2.01 (dt, 1H, *J* = 14.8, 4.4 Hz, one of CH_2_-2), 2.13 (ddd, 1H, *J* = 14.8, 11.5, 4.7 Hz, one of CH_2_-2), 2.25 (dd, 1H, *J* = 17.5, 3.0 Hz, one of CH_2_-7), 2.78 (dd, 1H, *J* = 17.5, 7.8 Hz, one of CH_2_-7), 2.84 (m, 1H, H-6), 4.36 (q, 1H, *J* = 7.0 Hz, H-1′); ^13^C-NMR (600 MHz, CDCl_3_) δ: 20.2 (C-3), 21.02 (C-4), 22.8 (C-2′), 28.0 (C-2), 28.7 (C-5), 33.0 (C-1′), 36.6 (C-7), 37.8 (C-6), 87.8 (C-1), 175.9 (C-8); IR (KBr, cm^−1^): 1774, 1173, 934; HRMS: (ESI-TOF) *m*/*z* [M + Na]^+^ calcd for C_10_H_15_IO_2_Na 317.0015; found 317.0018.

(+)-(1*R*,6*R*,1′*S*)-1-(1′-Iodoethyl)-9-oxabicyclo[4.3.0]nonan-8-one (**6b**), Obtained from acid **5b** (0.25 g, 1.5 mmol), yield 0.3 g (64%); Rt = 190 min, $\alpha_{D}^{25}$ = +58 (c = 0.45, CHCl_3_, ee > 99%); spectroscopic data identical to the values reported for (−)-(1*S*,6*S*,1′*R*)-**6a**

#### 2.3.7. 1-(1′-Bromoethyl)-9-Oxabicyclo[4.3.0]nonan-8-one (**7a** and **7b**)

##### General Procedure

To the unsaturated acid **5** dissolved in tetrahydrofuran, the solution of *N*-bromosuccinimide (1 g, 5.6 mmol) in tetrahydrofuran and a 0.1 mL of acetic acid were added. The reaction was stirred for two days at room temperature. Then, Et_2_O was added to the reaction and the mixture was washed with sodium bicarbonate and NaCl. The organic layer was dried over anhydrous MgSO_4_ and evaporated. The reaction product was purified on silica gel (hexane:acetone, 20:1). New 1-(1-bromoethyl)-9-oxabicyclo[4.3.0]nonan-8-one was obtained as the product. The physical and spectral data of synthesized bromolactone **7** are as follows:

(−)-(1*S*,6*S*,1′*R*)-1-(1′-Bromoethyl)-9-oxabicyclo[4.3.0]nonan-8-one (**7a**), Obtained from acid **5a** (0.5 g, 3 mmol), yield 0.5 g (67%); Rt = 186 min, $\alpha_{D}^{25}$ = −45 (c = 0.65, CHCl_3_, ee > 99%); mp 44–48 °C; ^1^H-NMR (600 MHz, CDCl_3_) δ: 1.26 (m, 1H, one of CH_2_-5), 1.34 (m, 1H, *J* = 13.9, 10.2, 3.6 Hz, one of CH_2_-4), 1.46 (dtt, 1H, *J* = 13.3, 11.2, 4.1 Hz, one of CH_2_-3), 1.60 (m, 1H, one of CH_2_-4), 1.66 (m, 1H, one of CH_2_-3), 1.72 (d, 3H, *J* = 6.8 Hz, CH_3_-2′), 1.83 (m, 1H, one of CH_2_-5), 1.95 (dt, 1H, *J* = 14.7, 4.5 Hz, one of CH_2_-2), 2.05 (ddd, 1H, *J* = 14.7, 11.6, 4.9 Hz, one of CH_2_-2), 2.25 (dd, 1H, *J* = 14.1, 2.5 Hz, one of CH_2_-7), 2.79 (dd, 1H, *J* = 14.1, 7.2 Hz, one of CH_2_-7), 2.81 (m, 1H, H-6), 4.19 (q, 1H, *J* = 6.8 Hz, H-1′); ^13^CNMR (600 MHz, CDCl_3_) δ: 20.0 (C-2′), 20.1 (C-3), 21.1 (C-4), 26.4 (C-2), 28.7 (C-5), 36.3 (C-6), 36.4 (C-7), 52.6 (C-1′), 87.9 (C-1), 176.1 (C-8); IR (KBr, cm^−1^): 2936, 1780, 1170, 974, 938, 641; HRMS: (ESI-TOF) *m*/*z* [M + H]^+^ calcd for C_10_H_15_BrO_2_ 247.0334; found 247.0335.

(+)-(1*R*,6*R*,1′*S*)-1-(1′-Bromoethyl)-9-oxabicyclo[4.3.0]nonan-8-one (**7b**), Obtained from acid **5b** (0.25 g, 1.5 mmol), yield 0.2 g (60%); Rt = 182 min, $\alpha_{D}^{25}$ = + 44 (c = 0.7, CHCl_3_, ee = 99%); spectroscopic data identical to the values reported for (−)-(1*S*,6*S*,1′*R*)-**7a**

#### 2.3.8. 1-(1′-Chloroethyl)-9-Oxabicyclo[4.3.0]nonan-8-one (**8a** and **8b**)

##### General Procedure

To the unsaturated acid **5** dissolved in tetrahydrofuran, the *N-C*hlorosuccinimide (0.78 g, 5.8 mmol) and 0,1 mL of acetic acid were added. The reaction was stirred for two days at room temperature. Afterwards, diethyl ether was added to the reaction and the mixture was washed with sodium bicarbonate and NaCl. The ether fraction was dried over anhydrous MgSO_4_ and evaporated. New 1-(1-chloroethyl)-9-oxabicyclo[4.3.0]nonan-8-one was obtained as the product. The physical and spectral data of synthesized chlorolactone **8** are as follows:

(−)-(1*S*,6*S*,1′*R*)-1-(1′-Chloroethyl)-9-oxabicyclo[4.3.0]nonan-8-one (**8a**), Obtained from acid **5a** (0.5 g, 3 mmol), yield 0.4 g (65%); Rt = 162 min, $\alpha_{D}^{25}$ = −41 (c = 0.4, CHCl_3_, ee = 99%); mp 37–40 °C; ^1^H-NMR (600 MHz, CDCl_3_) δ: 1.27 (m, 1H, one of CH_2_-5), 1.34 (m, 1H, one of CH_2_-4), 1.46 (m, 1H, one of CH_2_-3), 1.54 (d, 3H, *J* = 6.7 Hz, CH_3_-2′), 1.60 (m, 1H, one of CH_2_-4), 1.66 (m, 1H, one of CH_2_-3), 1.84 (m, 1H, one of CH_2_-5), 1.90 (dt, 1H, *J* = 14.8, 4.4 Hz, one of CH_2_-2), 1.97 (ddd, 1H, *J* = 14.8, 11.4, 4.9 Hz, one of CH_2_-2), 2.25 (dd, 1H, *J* = 17.2, 2.7 Hz, one of CH_2_-7), 2.74 (m, 1H, H-6), 2.79 (dd, 1H,*J* = 17.2, 7.8 Hz, one of CH_2_-7), 4.07 (q, 1H, *J* = 6.7 Hz, H-1′); ^13^C-NMR (600 MHz, CDCl_3_) δ: 19.3 (C-2′), 20.0 (C-3), 21.0 (C-4), 25.7 (C-2), 28.6 (C-5), 35.5 (C-6), 36.3 (C-7), 59.9 (C-1′), 88.0 (C-1), 176.1 (C-8); IR (KBr, cm^−1^): 1783, 1172, 976, 942; HRMS: (ESI-TOF) *m*/*z* [M + Na]^+^ calcd for C_10_H_15_ClO_2_Na 225.0658; found 225.0661.

(+)-(1*R*,6*R*,1′*S*)-1-(1′-Chloroethyl)-9-oxabicyclo[4.3.0]nonan-8-one (**8b**), Obtained from acid **5b** (0.25 g, 1.5 mmol), yield 0.2 g (63%); Rt = 159 min, $\alpha_{D}^{25}$ = +41 (c = 0.45, CHCl_3_, ee > 99%); spectroscopic data identical to the values reported for (−)-(1*S*,6*S*,1′*R*)-**8a**

### 2.4. Antimicrobial Activity of Lactones

The antimicrobial activity of racemic halolactones were tested towards three bacterial strains classified as Gram-negative (*Escherichia coli* ATCC 25922, *Pseudomonas aeruginosa* ATCC 27853, *Proteus mirabilis* ATCC 6380) and Gram-positive (*Bacillus cereus* ATCC 10876) and two fungal strains (*Candida albicans* ATCC 10231 and *Aspergillus brasiliensis* ATCC 16404). The microorganisms were supplied by Pasteur Institute of Algiers (Algeria).

The tests were carried out using the disc diffusion method. Briefly, overnight microbial cultures were adjusted to 0.5 MacFarland turbidity standards and spread in Petri dishes containing Mueller–Hinton and Sabouraud media for bacteria and fungi, respectively. The paper discs (6 mm diameter) was sterilized and impregnated with lactone dissolved in DMSO. Then, the discs were placed on the inoculated agar. The negative controls were discs immersed in pure DMSO. The plates were incubated for 24 h at 37 °C (bacteria) and for 48 h at 25 °C (yeast) and 5–7 days for fungi. Then, the inhibition zone diameters were determined in millimeters (including disc diameter of 6 mm). The test was carried out in triplicates.

### 2.5. Antiproliferative Activity

The GL-1 (canine B-cell leukemia) was obtained from Yasuhito Fujino and Hajime Tsujimoto from the Department of Veterinary Internal Medicine University of Tokyo [27], the CLB70 (canine B-cell chronic leukemia) was described earlier by Pawlak et al. [28]. The GL-1 cells were maintained in RPMI 1640 medium with 10% fetal bovine serum (FBS), 100 µg/mL streptomycin, 100 U/mL penicillin and 2 mM L-glutamine (Sigma-Aldrich, Stainheim, Germany). The CLB70 cell line was maintained in Advanced RPMI (Gibco) medium with 100 µg/mL streptomycin, 100 U/mL penicillin and 20% heat-inactivated FBS. Cells were cultured at 37 °C in the incubator with 95% humidified atmosphere and 5% CO_2_. The antiproliferative activity was tested according to the procedure described previously [17]. The tested lactones were prepared within a concentration range of 6.25–50 µg/mL in the culture medium (DMSO concentration was less than 1% in each dilution). The activity curves used to calculate IC_50_ are presented in the (Supplementary information Figures S7–S18). The optical density of formed formazan in untreated control cells was assigned as 100%. Viability of test samples was calculated as: % Viability = (average OD for test group/average OD for control group) × 100. The results were determined from more than 3 independent experiments (four wells each) and presented as mean IC_50_ value ± SD.

### 2.6. Hemolytic Activity

The hemolytic activity test was performed using pig red blood cells (RBCs) and their membranes (RBCMs). The method has been described previously by Pruchnik et al. [29]. The hemolytic activity of the lactones was tested as DMSO solutions (10, 20, 40, 60, 80, and 100 µM concentrations). The control contained only DMSO in the same volume as the tested samples. The final concentration of erythrocytes in the sample was 1.2% hematocrit. The measurement was recorded at 540 nm using a UV–Vis spectrophotometer (Specord 40, Analytik Jena, Jena, Germany). The percentage of hemolysis caused by the compounds was calculated based on the hemoglobin concentration released from red blood cells, according the following formula:

$$H\;\left(\%\right)\;=\;\frac{A_{s}}{A_{100\%}}\;\times 100\%$$

where *A*_s_ was absorbance of hemoglobin released to supernatant in probes, *A*_100%_ was defined as absorbance of hemoglobin released to supernatant in probes where RBCs were lysed in distilled water in 100%.

### 2.7. Fluorescence Spectroscopy

In the fluorometric method, the impact of six lactones (**6a**,**b**, **7a**,**b**, **8a** and **8b**) on red blood cell membranes (RBCMs) was investigated, especially, fluidity, hydration and packing arrangement of lipids. This analysis has been described precisely by Pruchnik et al. [29]. The RBCMs were isolated following the method presented earlier by Dodge et al. [30]. For fluorometric analysis, three fluorescent probes were applied: MC540 (Merocyanine 540), Laurdan (6-dodecanoyl-2-dimethylaminonaphthalene) and DPH (1,6-diphenyl-1,3,5-hexatriene) at the concentration in the samples of 1 µM. The excitation and emission wavelengths were as follows: for DPH, λ_exc_ = 360 nm and λ_em_ = 425 nm; for Laurdan, λ_exc_ = 360 nm and the emission wavelengths were λ_em_ = 440 nm and λ_em_ = 490 nm; and for MC540, λ_exc_ = 540 nm and λ_em_ = 585 nm.

The compounds were tested in the same concentrations as in the hemolytic activity test (10, 20, 40, 60, 80 and 100 µM). The control contained DMSO of the same volume as the tested samples. Measurements were carried out after 1 h of incubation at 37 °C, on a VARIAN CARY Eclipse fluorimeter, and performed in three independent experiments. The Statistica 13.0 was used for all statistical calculations. The results were analyzed by one-way ANOVA followed by Duncan test. Data are shown as mean values ± standard deviation (SD). *p* values ˂ 0.05 were considered statistically significant.

### 2.8. Fourier Transform Infrared Spectroscopy Method (FTIR)

The molecular interactions of the tested lactones with specific functional groups of lipid were investigated using the FTIR method. Lipid membranes have been used from RBCMs obtained from erythrocytes to serve as a model. The samples contained RBCMs and DMSO solution of lactones at final concentration 100 µM. The control samples contained the same amount of RBCMs as the tested samples and the addition of DMSO instead of lactones. After mixing, each sample was separately applied at the ZnSn plate. The spectrum of samples (with water) were taken at 37 °C using a Thermo Nicolet 6700 MCT (Thermo Fisher Scientific, Waltham, MA, USA). To remove the water, the plate was incubated for 24 h at 37 °C. After drying the samples, measurements were repeated. In the spectrum of the tested samples, we considered the RBCMs’ characteristic bands.

## 3. Results and Discussion

### 3.1. Synthesis of Halolactones

**Scheme 1:**
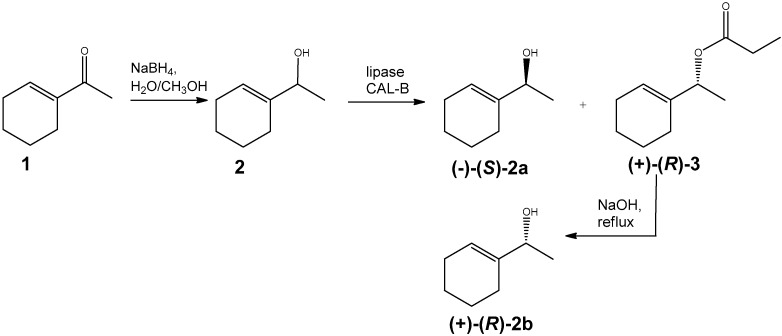
Synthesis of enantiomeric 1-(cyclohex-1-en-1-yl) ethanol (**2a** and **2b**).

**Scheme 2:**
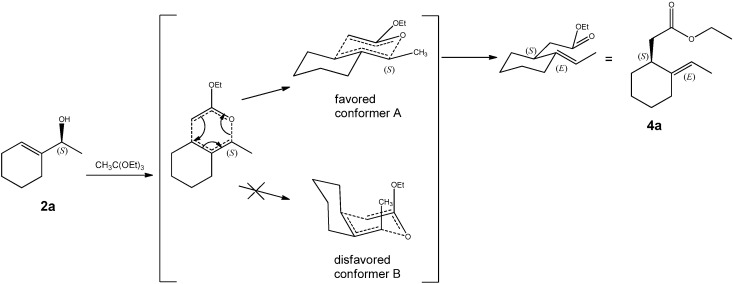
The mechanism of Johnson–Claisen rearrangement of (*S*)-1-(cyclohex-1-en-1-yl) ethanol **2a**.

**Scheme 3:**
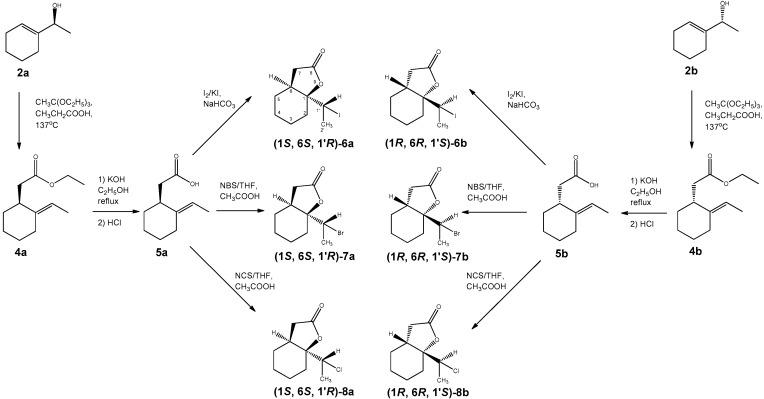
Synthesis of enantiomeric pairs of halolactones **6**, **7**, **8** from (*S*)-1-(cyclohex-1-en-1-yl) ethanol **2a** and (*R*)-1-(cyclohex-1-en-1-yl)ethanol **2b**.

The halolactones (**6**–**8**) were obtained in five steps of chemical synthesis (Scheme 1, Scheme 2 and Scheme 3) and together with all intermediates (**2**–**5**) their structures were established on the basis of spectroscopic data (IR, ^1^H NMR, ^13^C NMR, COSY, HMQC, HMBC). Starting from 1-acetylcyclohexene (**1**), the standard reduction with sodium borohydride in methanol was performed to obtain the racemic allylic alcohols (**2**). The next step was kinetic resolution of alcohol **2**. The reaction was performed in diisopropyl ether, catalysed by lipase CAL-B from *Candida antarctica*; vinyl propionate was used as a acyl donor. As a result, the mixture of (*S*)-alkohol (**2a**) and (*R*)-enantiomer of propionate (**3**) was afforded. The configuration of stereogenic centres was assigned on the basis of specific optical rotation data presented in the literature [21,22]. The obtained results are in accordance with our previous findings for 4-arylbut-3-en-2-ols and the Kazlauskas’ rule, which assume the enantiopreference of most lipases for transersryfication of (*R*)-enantiomer of alcohols [31,32]. A prerequisite is a significant difference in size of substituent at the stereogenic centre and the following sequence of substituents: OH > large substituent > medium substituent. After kinetic resolution, the products were separated on column chromatography and the (*R*)-enantiomer of propionate was hydrolysed in basic conditions to (*R*)-alkohol (**2b**).

Two enantiomers of alcohol **2** were subjected to Johnson–Claisen rearrangement which provides transfer of chirality to the corresponding esters as shown on Scheme 2 for rearrangement of (*S*)-alkohol **2a**. The careful analysis of the mechanism of this rearrangement allowed as to study the configurations of stereogenic centers in esters (**4a** and **4b**). One can see that the 1,3-diaxial interaction in chairlike transition state disfavor the formation of conformer B. Favorable formation of the conformer A leads to ester **4a** with *S*-configuration on the new stereogenic center. This is in accordance with previous findings that for the alcohols with the *E*-configuration of double bond the retention of configuration is observed [33,34]. Therefore, from (*E*,*S*)-alcohol **2a** the (*E*,*S*)-ester **4a** was formed and from (*E*,*R*)-alcohol **2b** the (*E*,*R*)-ester **4b** was obtained. Afterwards, the esters were hydrolyzed in ethanolic NaOH solutions. The alkaline hydrolysis does not affect configuration of stereogenic centers and hence, the (*S*)-acid **5a** and (*R*)-acid **5b** were obtained, respectively, from ester **4a** and **4b**.

The incorporation of different halogen atoms was possible with the application of different halolactonization strategies. Starting from enantiomers of unsaturated acids (**5a** and **5b**), iodo- bromo- and chlorolactonisation were performed. For the iodolactonization reaction, the iodine and potassium iodide in the biphasic Et_2_O/NaHCO_3_ system were used. For bromo- and chlorolactonisation, the process was carried out in tetrahydrofuran with corresponding *N*-bromosuccinimide or *N*-Chlorosuccinimide, respectively, and in the presence of a catalytic amount of acetic acid. In each halolactonization reaction the corresponding δ-halo-γ-lactone was obtained as the only product (Scheme 3).

**Scheme 4:**
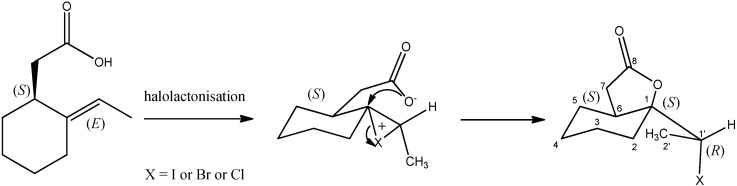
The mechanism of halolactonisation of (*S*)-acid **5a** and the resulting configuration of stereogenic centers in halolactones **6a**, **7a** and **8a**.

The mechanisms of different halolactonisations reactions involves the formation of positively charged halonium cations, as well as the activation of the carboxyl group to a carboxylate anion. The nucleophilic attack of an oxygen atom from carboxylate ion is highly stereospecific and goes from opposite sides of halonium ion. The steric consequences are the antiperiplanar orientations of C-X and C-O bonds [17,19,35,36] that determine configurations of two new stereogenic centers at C-1 and C-1′ in the formed halolactones. The rigid structure of the cyclohexane ring as well as the presented lactonisation mechanisms allowed us to assign (1*S*,6*S*,1′*R*) configuration for halolactones **6a**, **7a** and **8a** obtained from (*S*)-acid **5a** (Scheme 4). Analogically, the lactonisation of (*R*)-enantiomer of acid **5b** results in the formation of opposite enantiomers **6b**, **7b**, **8b** with *R*-configuration on C-1 and C-6 and *S*-configuration on C-1′ carbon atom.

The IR spectra of iodolactones (**6a** and **6b**) showed the presence of absorption bands at 1774 cm^−1^ characteristic for a γ-lactone ring. In the ^1^H-NMR spectrum quartet (*J* = 7.0 Hz), the H-1′ proton is present at 4.36 ppm. The same coupling constant can be found in the doublet from three protons at 1.94 ppm. The shift of those signals towards the lower field is caused by the deshielding effect of a iodine atom. The protons of the methylene group C*H*_2_-7 (2.25 and 2.78 ppm) are shown as pair of doublets of doublets coupled with a large geminal coupling constant (*J* = 17.5 Hz). The signals present on ^13^C NMR spectrum also confirm the formation of γ-lactone witch iodoethyl substituent (**6a** and **6b**). The signal from the carbon atom C-1′ is present at 33.0 ppm and from C-1 at 87.8 ppm. The COSY, HSQC and HMBC correlations were useful tool for assignment the signals on ^1^H-NMR spectrum to the corresponding methylene protons in cyclohexane ring. Table 1 present the sequence of correlation observed on HMBC spectrum. The crucial ones were coupling between C-1′ and C*H*_2_-2 and between C-6 and C*H*_2_-4 and C*H*_2_-5.

The ^1^H NMR spectra of bromo- and chlorolactones are almost identical compared to iodolactone in terms of the signal shape and chemical shift value. The main differences relate to ^13^C-NMR, where the complex effect on chemical shifts caused by halogen atoms can be observed. The correlation between halide electronegativity and ^13^C shift for the α-carbons is clearly seen on the spectra of three lactones. Usually, the effects of halogen atoms directly bonded to carbon follow the electronegativities of the elements in series chlorine to bromine. The iodine atom is an exception due to the “heavy atom effect” related to increased diamagnetic shielding caused by the large number of electrons derived from iodine. This tendency can be seen for C-1′ carbon atom of iodolactone **6a**,**b** shifted to upper field (33.0 ppm) in comparison to bromo- and chlorolactone (52.6 and 59.9 ppm respectively). The bond polarization due to the electronegativity of halogens should propagate along the carbon chain but there is an opposite correlation between the substituent electronegativity and chemical shifts of C-2′ carbon. As the inductive effects play a predominant role, the β-carbons are slightly shifted downfield on all halolactones spectra [37].

### 3.2. The Biological Activity

The screening of antimicrobial properties of synthesized halolactones was performed on four bacterial strains, a fungal strain and a yeast strain. The diffusion test aimed at pre-screening antimicrobial properties and was performed on racemic compounds **6**, **7**, **8**. If the compounds showed high activity; we would plan to determine it also for enantiomers. However, the activity towards bacterial strains was not significant, therefore, further in-depth study seemed to be unfounded. The tested halolactones was not active against yeast and fungal strains *Candida albicans* ATCC 10231 and *Aspergillus brasiliensis* ATCC 16404 as well as the Gram-negative bacteria *Pseudomonas aeruginosa ATCC 27853*. The tested iodo- and bromolactones (*rac*-**6** and *rac*-**7**) exhibited rather moderate antibacterial activity towards Gram-positive bacteria *Bacillus cereus* ATCC 10876 (inhibition zone 12 ± 0.00 and 8 ± 0.00 mm respectively) and Gram-negative *Escherichia coli* ATCC 25922 (inhibition zone 7 ± 0.00 and 9 ± 0.00 mm respectively). The most sensitive strain was Gram-negative *Proteus mirabilis* ATCC 35659, the diameter of inhibition zone was 11 ± 0.00 mm for iodolactone **6**, 12 ± 0.35 mm for bromolactone **7** and 12 ± 0.00 mm for chlorolactone **8**. A similar tendency, have also been noticed by Grabarczyk and coworkers [38,39,40]. In previous research, γ-lactones with similar carbon skeleton exhibited higher selectivity towards bacterial strains than towards yeast or fungal strains. In general, the most responsive were the Gram-positive bacteria like *B. cereus* and Gram-negative *E. coli*.

The antyproliferative activity of lactone enantiomers was tested on two canine cell lines CLB70 and GL-1 (Table 2). The choice of these specific cell lines was due to the possibility of comparing the results to previous experiments [18,19,20]. All tested substances exhibited significant activity. The GL-1 cell line was more sensitive to all tested compounds including the control etoposide. For the CLB70 cell line, the small differences in activity can be observed between the enantiomer activities, especially comparing the more potent (−)-enantiomer of bromolactone (**7a**) to less active (+)-enantiomer **7b**. Overall, there were no significant differences in lactone activity regardless of halogen type and configuration of stereogenic centers. It is worth to point that the activity of tested compounds was comparable with the control and was much higher than antiproliferative activity of bicyclic lactones with the cyclohexane system reported earlier [20,41].

**Figure 1:**
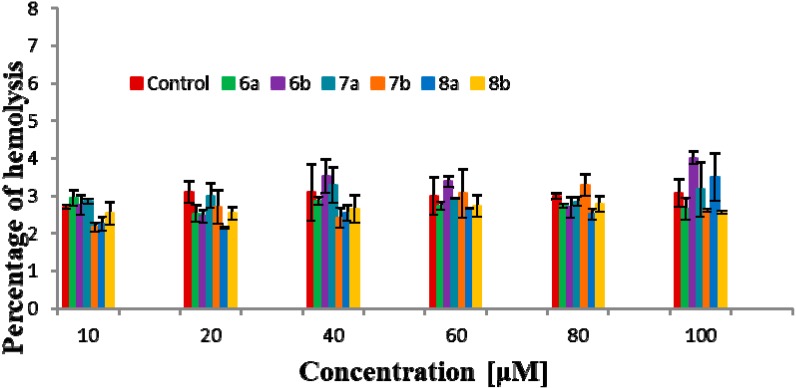
Hemolysis of RBCs dependent on concentration of compounds.

The cytotoxicity of compounds was evaluated on the basis of hemolytic test. The results presented as the percentage of hemolysis are summarized on Figure 1. The percentage of hemolysis registered for all lactones was not significantly different from the percentage of hemolysis in control samples. Even at much higher concentrations (1 mM), the compounds showed no hemolytic activity (Table S1). Therefore, the compounds did not induce an increase in hemolysis in tested concentrations. According to toxicity classification, a compound is nontoxic if the hemolysis does not exceed 9% [42]. In this regard all tested compounds did not have a toxic effect on red blood cells (RBCs). These results are consistent with the results obtained for bromolactones with a 2,5-dimethylfenyl substituent [19].

### 3.3. Biophysical Research

The interactions of lactones with red blood cell membranes (RBCMs) were studied using fluorescence spectroscopy and Fourier transform infrared spectroscopy (FTIR) methods. The fluorescence spectroscopy allowed us to measure effects of tested compounds on physicochemical properties of RBCMs, especially fluidity, hydration and packing arrangement of lipids in membranes. Three fluorescent probes (DPH, Laurdan and MC540) were used in those experiments. Each of these probes is located in a different area of the membrane. The DPH probes are located in hydrophobic region of the membrane. Laurdan is incorporated at the level of the phospholipid glycerol backbone (hydrophilic-hydrophobic regions) and MC540 is attached above the glycerol skeleton.

**Figure 2:**
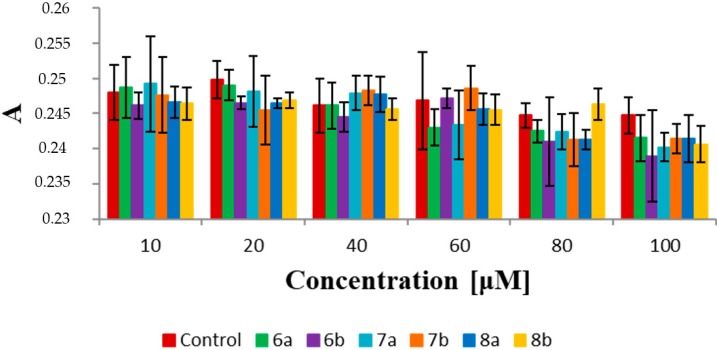
Values of anisotropy (A) of DPH probe for RBCMs (control) and RBCMs with addition of compounds **6a**,**b**, **7a**,**b**, **8a**,**b** at 37 °C. Values are mean ± SEM. Values are not significantly (*p* ˂ 0.05) different from control.

The DPH results are presented as the changes in fluorescence anisotropy (A) and provide information about fluidity of the hydrocarbon chains in the phospholipid bilayers (Figure 2). The decrease in the value of anisotropy determines increased fluidity of the membrane and the increased anisotropy indicates increased organisation of hydrocarbon chains and, hence, the rigidity of the membrane. The results showed that the presence of compounds does not change the value of anisotropy. This suggests that all bicyclic δ-halo-γ-lactones did not have an impact on fluidity in the hydrophobic region of the lipid bilayers.

**Figure 3:**
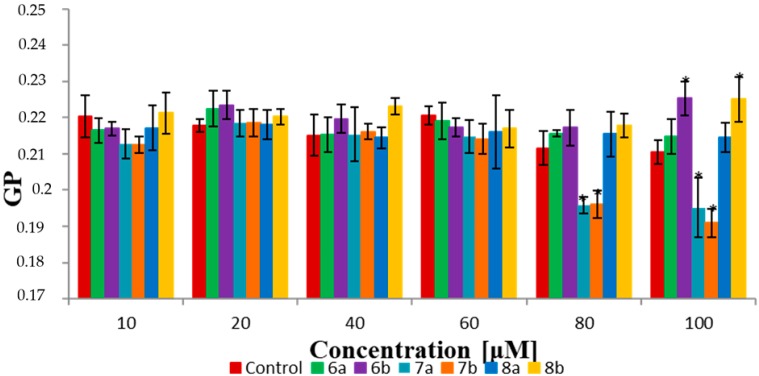
Values of generalized polarization (GP) of Laurdan probe for the RBCMs (control) and RBCMs with addition of compounds **6a**,**b**, **7a**,**b**, **8a**,**b** at 37 °C. Values are mean ± SEM. Means labeled with asterisk (*) are significantly (*p* ˂ 0.05) different from control.

Laurdan locates in the hydrophilic–hydrophobic interface of the bilayer. This fluorescent probe is sensitive to polarity modifications and reflects changes in hydration and packing order in the head groups of lipids in RBCMs [43,44]. The interactions of lactones with membranes were determined on the basis of generalized polarization (GP). The results indicate small changes in GP values for all bicyclic δ-halo-γ-lactones in the highest concentrations (80 and 100 μM). For two enantiomeric pairs (**6a** and **6b**, **8a** and **8b**), a slight increase of GP values can be observed (Figure 3). In both cases, the higher values were registered for (+)-enantiomers (**6b** and **8b**). It was probably indicated by an increase in the organization in hydrophobic–hydrophilic regions of the phospholipid bilayers which was associated with a decrease in water content in the area of glycerol backbone. Interestingly, for **7a** and **7b,** the effect was the opposite. The results have shown a small decrease in GP values at 80 and 100 μM concentrations. It indicated that more water molecules were incorporated at the level of the glycerol backbone, consequently, decreasing the order in the polar group of lipids. These results suggest that bromolactones **7a** and **7b** are probably located slightly deeper in the hydrophilic area than iodo- and chlorolactones.

**Figure 4:**
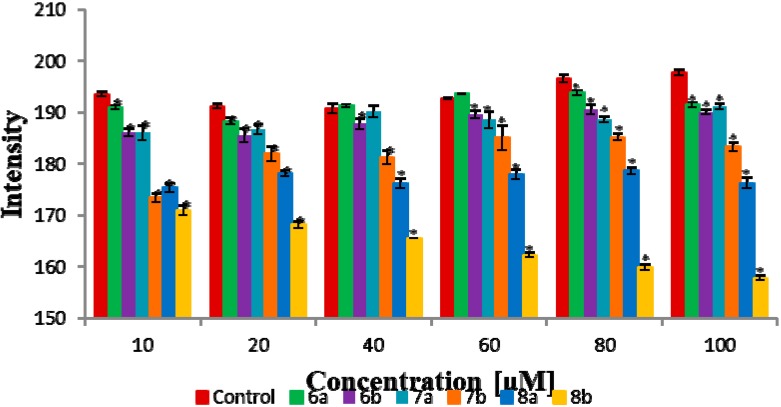
Changes of intensity of MC540 probe measured at 585 nm for RBCMs (control) and RBCMs with addition of tested compounds. The measurement was carried out at 37 °C. Values are mean ± SEM. Means labeled with asterisk (*) are significantly (*p* ˂ 0.05) different from control.

The MC540 probe is located slightly higher in the membrane than Laurdan. This probe is very sensitive to lipid packing in the membrane and aqueous environments [45,46,47]. The fluorescence intensity of MC540 strongly increases in the liquid phase of lipid bilayers and decreases in the gel phase. In the presented experiments, the maximum of fluorescence intensity (585 nm) was recorded for each sample and compared to the control (Figure 4). The results have shown a small decrease in fluorescence intensity registered in experiments with all bicyclic δ-halo-γ-lactones. The largest decrease was observed for the chlorolactone **8b**. The results suggest that in the presence of **8b**, the organization of the lipids in bilayers probably increases.

Generally, fluorescence spectroscopy results have shown that the compounds are weakly incorporated into the membrane. Only weak changes in the hydrophilic area were observed.

**Figure 5:**
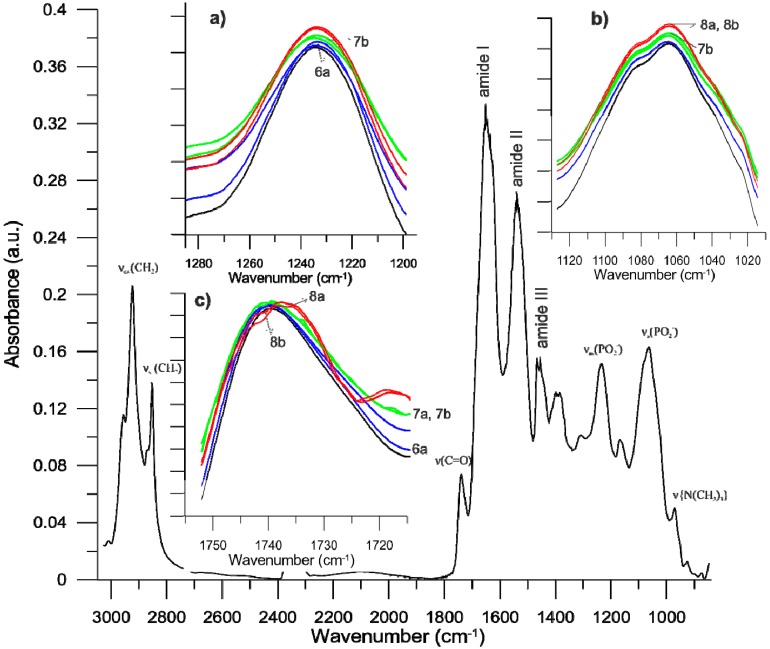
FT-IR spectra of erythrocyte membrane RBCMs (black lines) and of RBCMs modified with lactones (blue lines—**6a** and **6b**, green lines—**7a** and **7b**, red lines—**8a** and **8b**); (**a**) phosphate band I (asymmetric vibration), (**b**) phosphate band II (symmetric vibration), (**c**) carbonyl band.

Spectroscopic studies in the near infrared band allowed to register absorption spectra for isolated red blood cell membranes (RBCMs). In the IR spectra, the characteristic frequency bands of the protein and lipid components were analyzed (Figure 5). We identified the distinguished erythrocyte frequency bands for individual membrane components: methyl groups and methylene hydrocarbon chains (2980–2830 cm^−1^), carbonyl groups of lipids (1760–1710 cm^−1^), the amide band I (80% C=O stretch, near 1650 cm^−1^), amide band II (60% N-H bend and 40% C-N stretch, near 1550 cm^−1^), amide band III (40% C-N stretch, 30% N-H bend, near 1300 cm^−1^), phosphate band (1280–1020 cm^−1^) and choline band (1000–940 cm^−1^) [48,49].

Figure 5 shows the infrared spectra of RBCMs and RBCMs in the presence of 100 µM of **6a**, **6b**, **7a**, **7b**, **8a** and **8b** compounds. Analysis of the IR spectra of RBCMs treated with six lactones showed no significant changes in the hydrophobic part of the membrane because none of the tested compounds change the frequency of signal for hydrocarbon chains (Figure S19). There were also no changes in the vibrational bands of choline groups of lipids, but they appeared within the bands of carbonyl and phosphate groups (Table S2). The carbonyl and phosphate groups form hydrogen bonds with water. Therefore, changes observed in these bands testify to changes in the degree of hydration of the carbonyl and phosphate groups [50,51]. A slight frequency shifts of the ester groups we observed the presence of lactones, in particular for **7b**, **8a** and **8b**, which suggests that these compounds change hydration in the area of lipid carbonyl groups (Figure 5c). Similar changes were observed in the polar regions of the lipid bilayers. The interaction between lactones and the head group of RBCMs was monitored by analyzing the symmetric and asymmetric phosphate bands. An increase in the wave number of the maximum of this band is indicative of the location of the lipid phosphate polar head group in less polar environments and a decrease in head group hydration (or hydrogen bonding) [49,52,53]. The tested compounds shifted the maximum of the phosphate band very slightly towards lower values of wave numbers, especially **7b**, **8a** and **8b** for ν_s_(PO_2_^−^) (Figure 5b) and **6a**, **7b**, **8a** and **8b** for ν_as_(PO_2_^−^) (Figure 5a). Additionally, in the presence of lactones the band of the phosphate group is slightly broader than for pure RBCMs, indicating that there might be an interaction between the investigated compound and the lipid [29]. In addition, preliminary analysis of the amide bands indicates that the tested compounds slightly affect the structure of proteins present in the membrane, which is the subject of our next work.

The results obtained from IR tests confirm the changes observed in the fluorimetric method. The tested compounds do not change the fluidity of RBCMs in the hydrophobic region of the lipid bilayer; they slightly affect the degree of lipid packing and the associated hydration of the RBCMs’ polar membrane. Interestingly, there are slight differences in the impact on these parameters depending on the type of halogen in the lactone structure. Larger changes in RBCMs are observed for chloro- and bromolactones, probably these compounds interact more strongly with the polar part of the lipid bilayer. However, we do not observe significant changes within the enantiomeric pairs of bicyclic δ-halo-γ-lactones.

## 4. Conclusions

The chemoenzymatic synthesis was applied to obtain new biologically active δ-halo-γ-lactones. The lipase CAL-B-catalyzed kinetic resolution was the crucial step leading to separation of 1-(cyclohex-1-en-1-yl) ethanol enantiomers and consequently allowed us to obtain opposite stereoisomers of lactones. Analyzing the mechanism of Johnson–Claisen rearrangement and the halolactonization, we were able to predict the configurations of stereogenic centers in the obtained compounds. As a result, pairs of enantiomerically pure (ee ≥ 99%) halolactones containing iodine, bromine or chlorine atom were synthesized. All tested compounds exhibited significant antiproliferative activity, being slightly more active against acute leukemia (GL-1) than a chronic leukemia (CLB70) cell line. Importantly, we did not observed much influence of halogens and configuration of chiral centers on this activity. The studies on interactions of lactones with red blood cell membranes revealed that tested compounds cause weak changes in the hydrophilic area of bilayer whereas no influence on fluidity in the hydrophobic region was observed. The presented results demonstrated that the tested lactones have a tendency for weak binding into the membrane but there is no evidence on increasing membrane permeability. Increased permeability may lead to cell lysis or, alternatively, may provide small changes in the lipid bilayer function that entail cell death. Taking into consideration that halolactones were non-toxic to erythrocytes in the hemolysis test, more in-depth studies are required to explain the mechanism of their promising antiproliferative activity.

## Figures and Tables

**Table 1:** Heteronuclear multiple-bond correlation—spectroscopy data of iodolactone **6**.

	CH_2_-2	CH_2_-3	CH_2_-4	CH_2_-5	H-6	CH_2_-7	H-1′	CH_3_-2′
C-1	+	+		+		+	+	+
C-2		+	+				+	
C-3			+	+				
C-4		+		+				
C-5			+		+	+		
C-6			+	+		+	+	
C-7				+				
C-8					+	+		
C-1′	+				+			+
C-2′							+	

**Table 2:** The antiproliferative activity of halolactones **6a**,**b**, **7a**,**b**, **8a** and **8b** and control—Etoposide against the selected cancer cell lines expressed as IC_50_.

Cell Line	Compound (IC_50_ µg/mL)						
	6a	7a	8a	6b	7b	8b	Etoposide
CLB70	19.59 ± 0.51	19.40 ± 0.35	23.93 ± 5.14	19.93 ± 0.07	28.57 ± 4.89	18.43 ± 1.46	14.31 ± 2.83
GL-1	13.76 ± 1.08	15.55 ± 0.79	17.17 ± 3.62	14.53 ± 2.38	14.95 ± 1.93	11.40 ± 0.40	4.4 ± 1.14

## Supplementary Materials

The following are available online at https://www.mdpi.com/2218-273X/10/1/95/s1, Figure S1: ^1^H-NMR of lactone 6, Figure S2: ^13^C-NMR of lactone 6, Figure S3: ^1^H-NMR of lactone 7, Figure S4: ^13^C-NMR of lactone 7, Figure S5: ^1^H-NMR of lactone 8, Figure S6: ^13^C-NMR of lactone 8, Figure S7: Dose-response curve used to calculate IC_50_ for lactone **6a** and CBL70 cell line, Figure S8: Dose-response curve used to calculate IC_50_ for lactone **6a** and GL-1 cell line, Figure S9: Dose-response curve used to calculate IC_50_ for lactone **7a** and CBL70 cell line, Figure S10: Dose-response curve used to calculate IC_50_ for lactone **7a** and GL-1 cell line, Figure S11: Dose-response curve used to calculate IC_50_ for lactone **8a** and CBL70 cell line, Figure S12: Dose-response curve used to calculate IC_50_ for lactone **8a** and GL-1 cell line, Figure S13: Dose-response curve used to calculate IC_50_ for lactone **6b** and CBL70 cell line, Figure S14: Dose-response curve used to calculate IC_50_ for lactone **6b** and GL-1 cell line, Figure S15: Dose-response curve used to calculate IC_50_ for lactone **7b** and CBL70 cell line, Figure S16: Dose-response curve used to calculate IC_50_ for lactone **7b** and GL-1 cell line, Figure S17: Dose-response curve used to calculate IC_50_ for lactone **8b** and CBL70 cell line, Figure S18: Dose-response curve used to calculate IC_50_ for lactone **8b** and GL-1 cell line, Figure S19: FT-IR spectra of erythrocyte membrane RBCMs (black lines) and of RBCMs modified with lactones (blue lines—**6a** and **6b**, green lines—**7a** and **7b**, red lines—**8a** and **8b**) for symmetric and asymmetric CH_2_ stretching band, Table S1: Hemolysis of RBCs dependent on concentration of compounds, Table S2: Selected bands (wavenumbers cm^−1^) of IR spectra of RBCM and RBCM+compounds (100 μM).
