# Supplementary material for: Chemoenzymatic Synthesis of Enantiomeric, Bicyclic δ-Halo-γ-lactones with a Cyclohexane Ring, Their Biological Activity and Interaction with Biological Membranes
Source: Biomolecules. 2020 Jan 6;10(1):95. doi: 10.3390/biom10010095 (PMC7022392; doi:10.3390/biom10010095)
Supplement: Supplementary file 1 [file biomolecules-10-00095-s001.pdf]

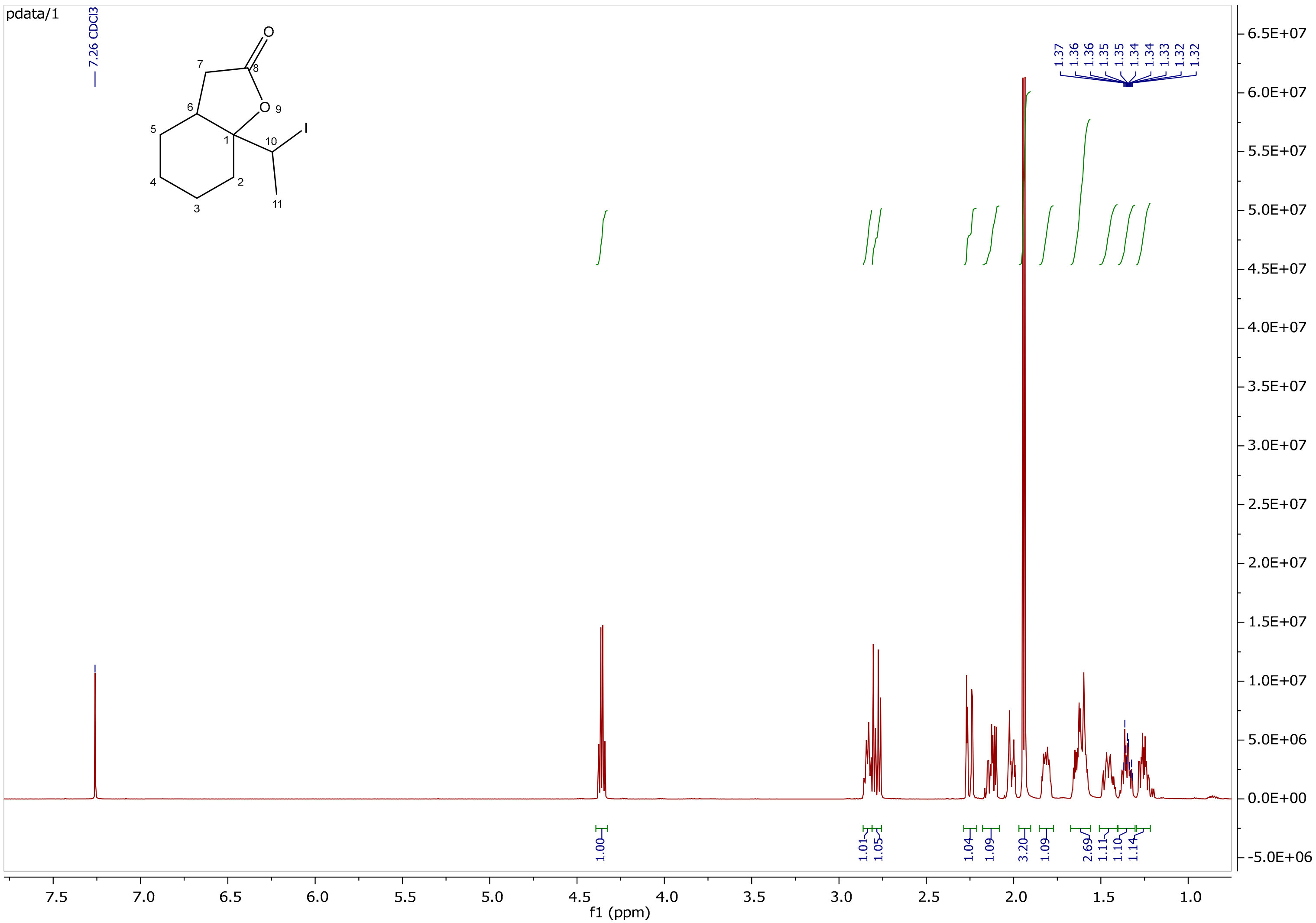

Figure S1: ^{1}H-NMR of lactone 6

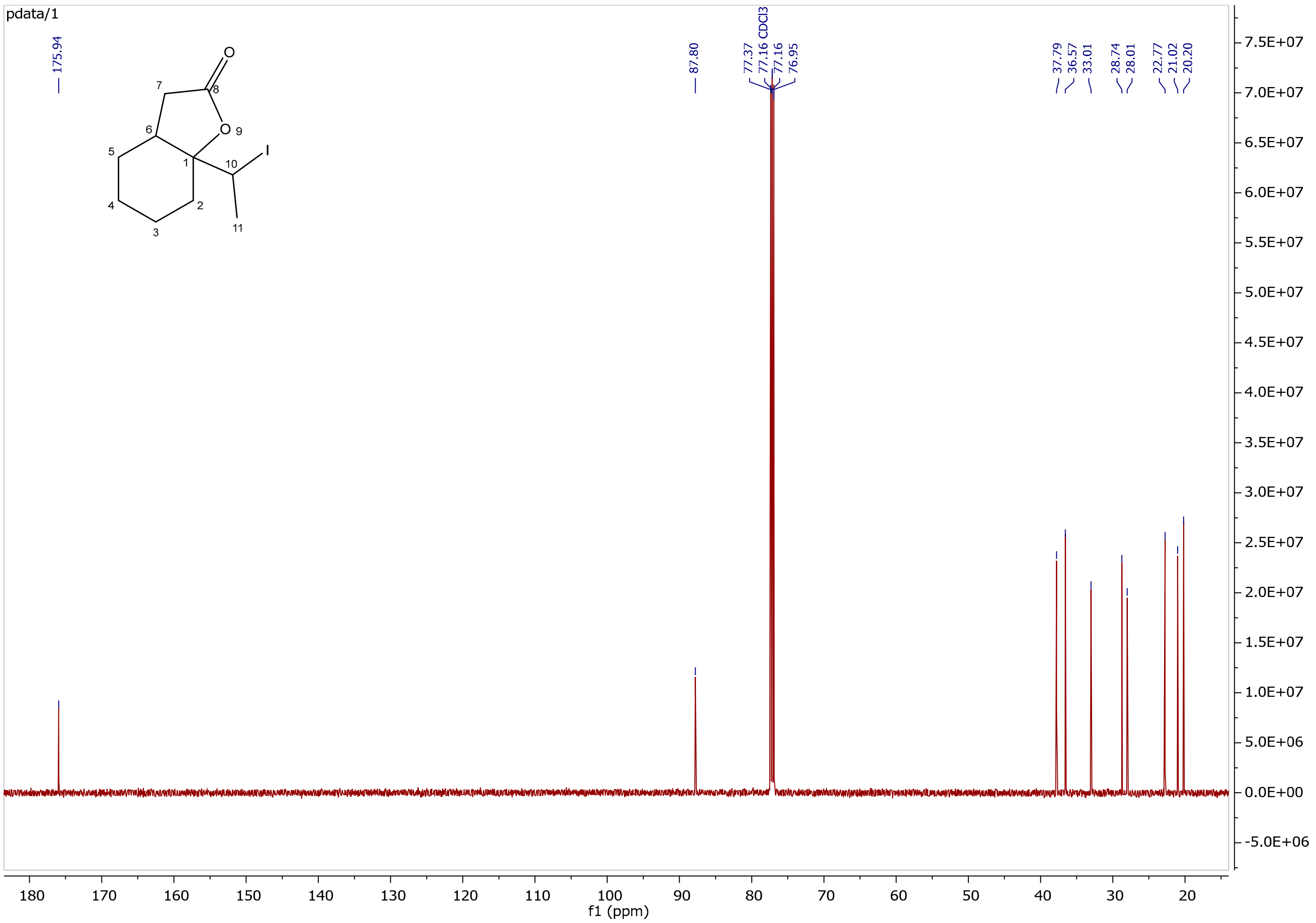

Figure S2: ^{13}C-NMR of lactone 6

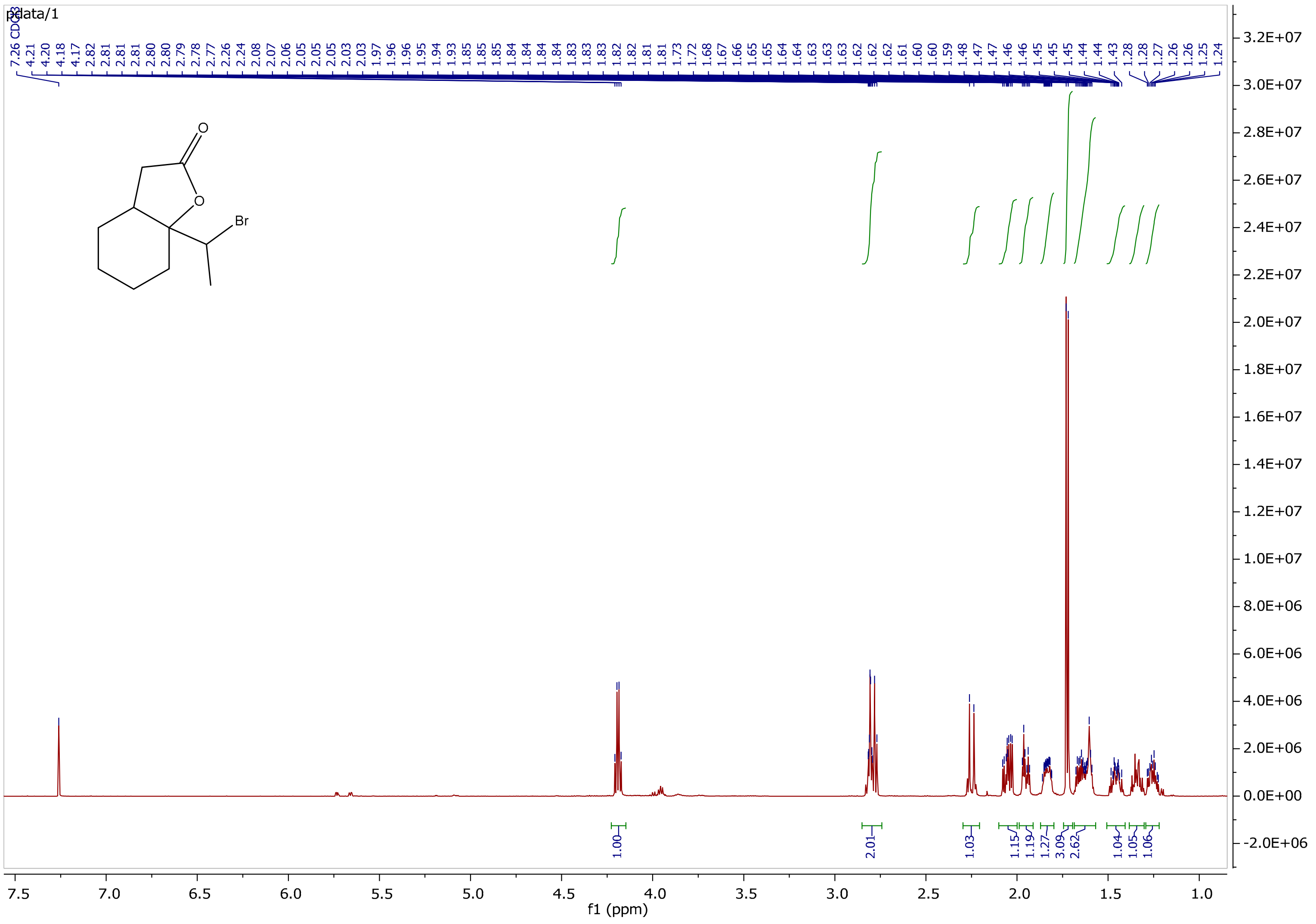

Figure S3: ^{1}H-NMR of lactone 7

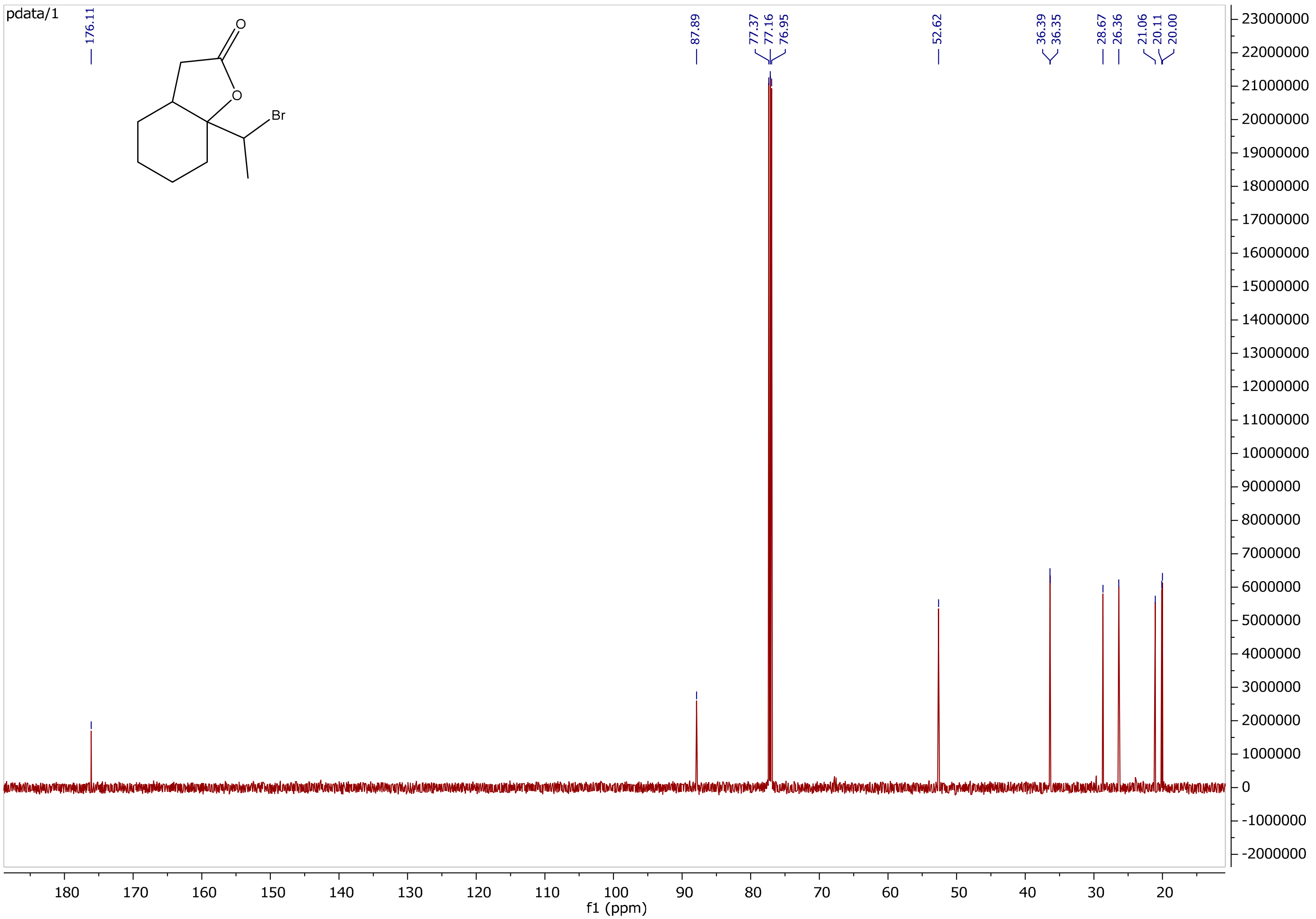

Figure S4: ^{13}C-NMR of lactone 7

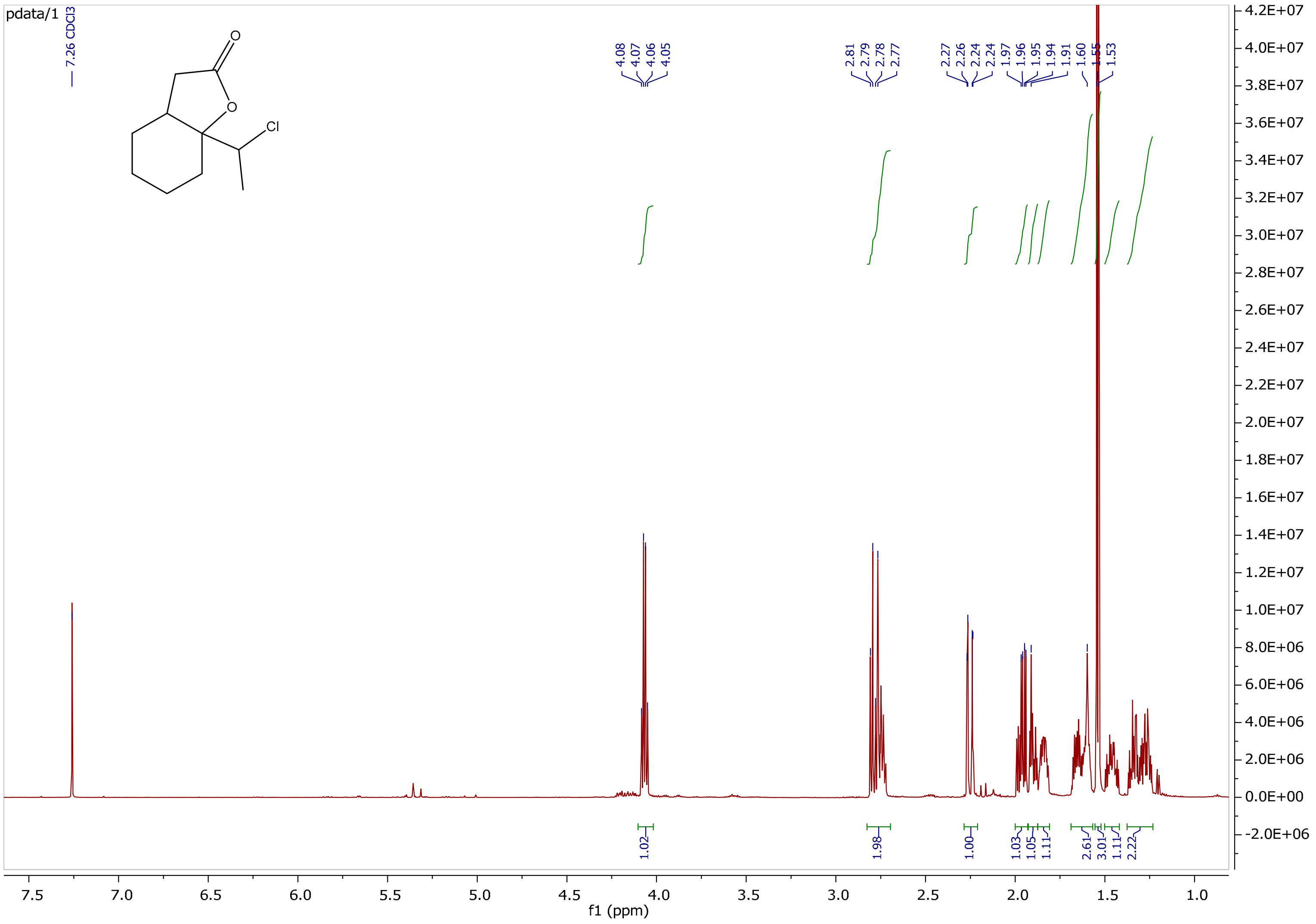

Figure S5: ^{1}H-NMR of lactone 8

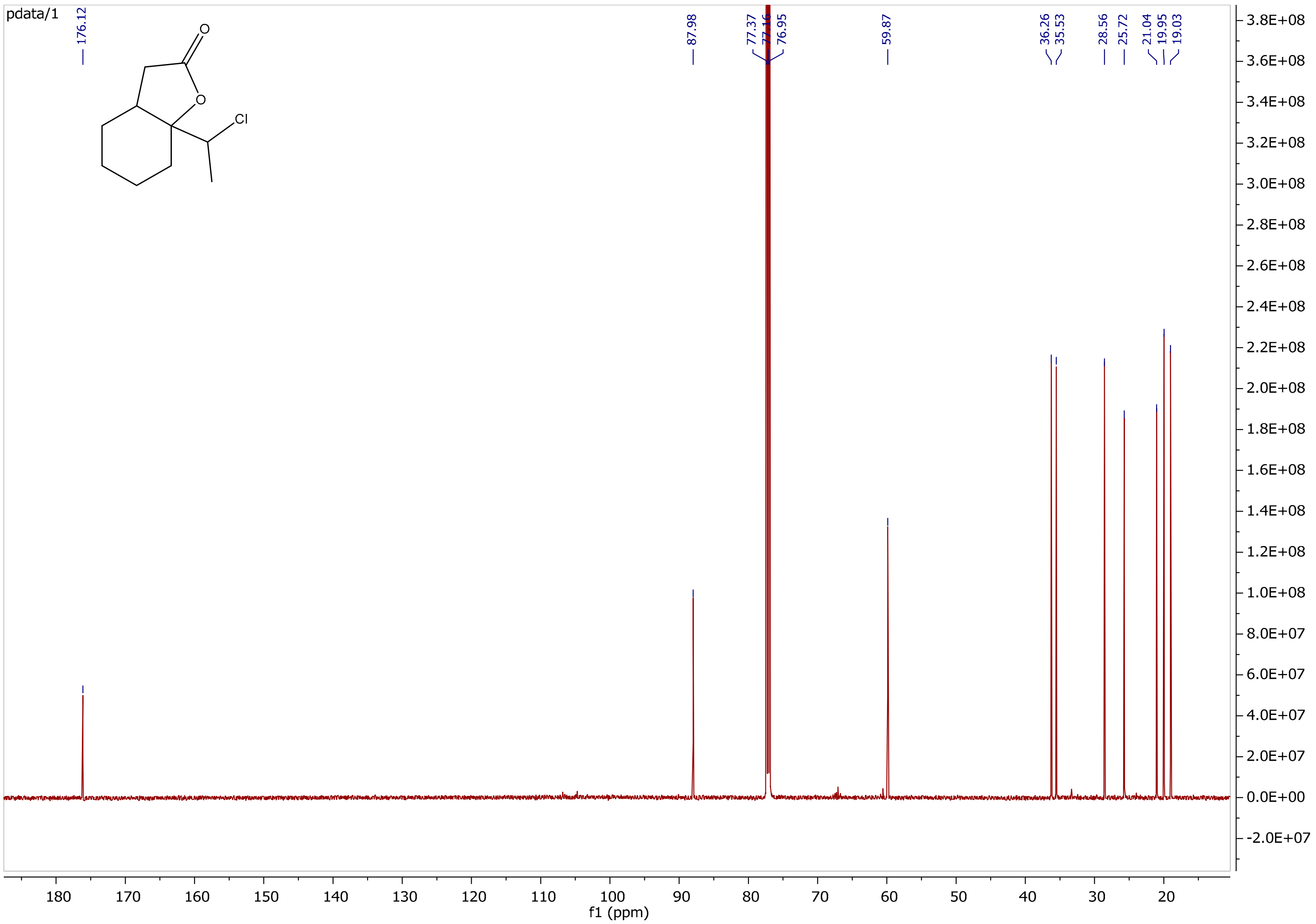

Figure S6: ^{13}C-NMR of lactone 8

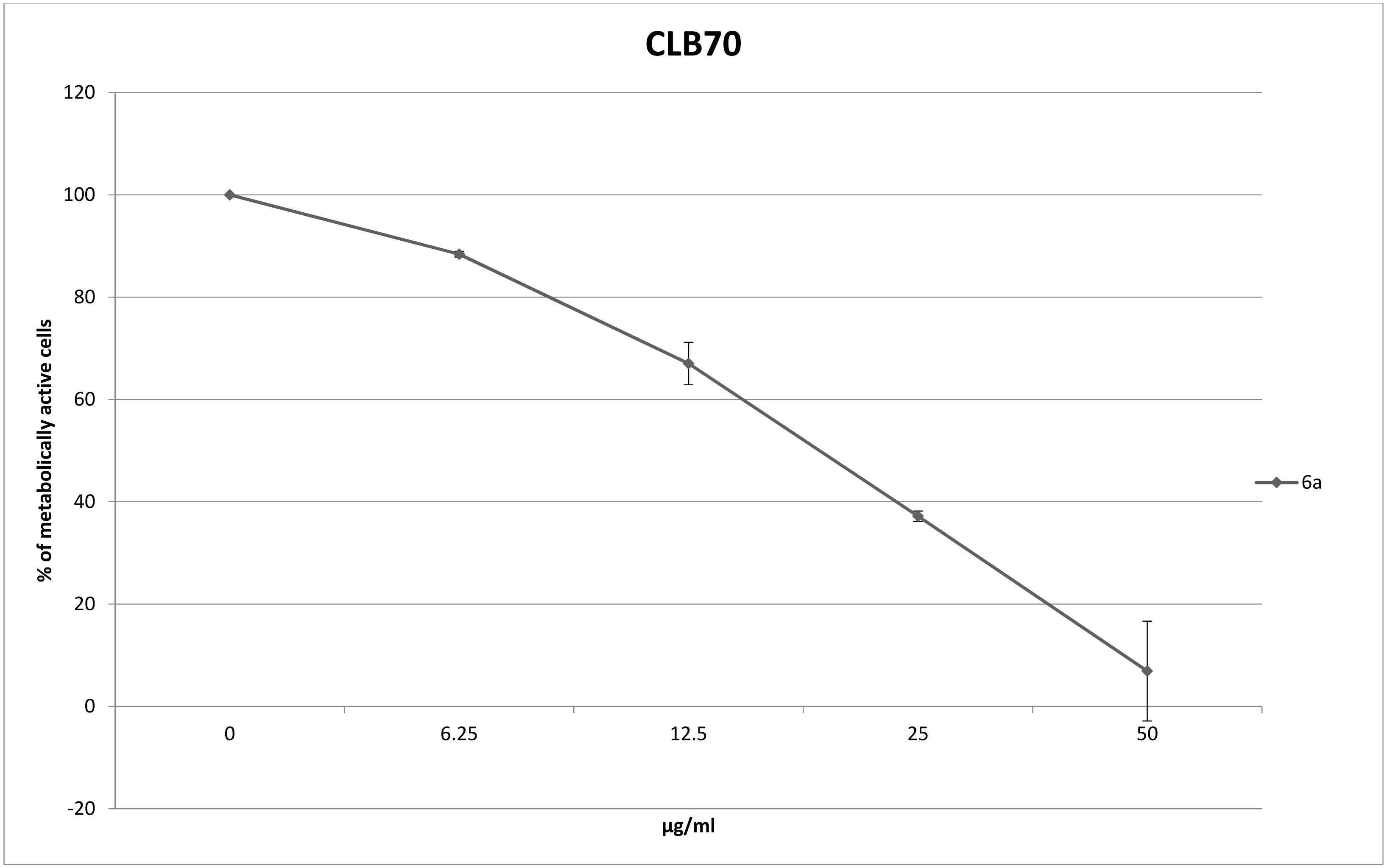

Figure S7: Dose-response curve used to calculate IC_{50} for lactone **6a** and CBL70 cell line.

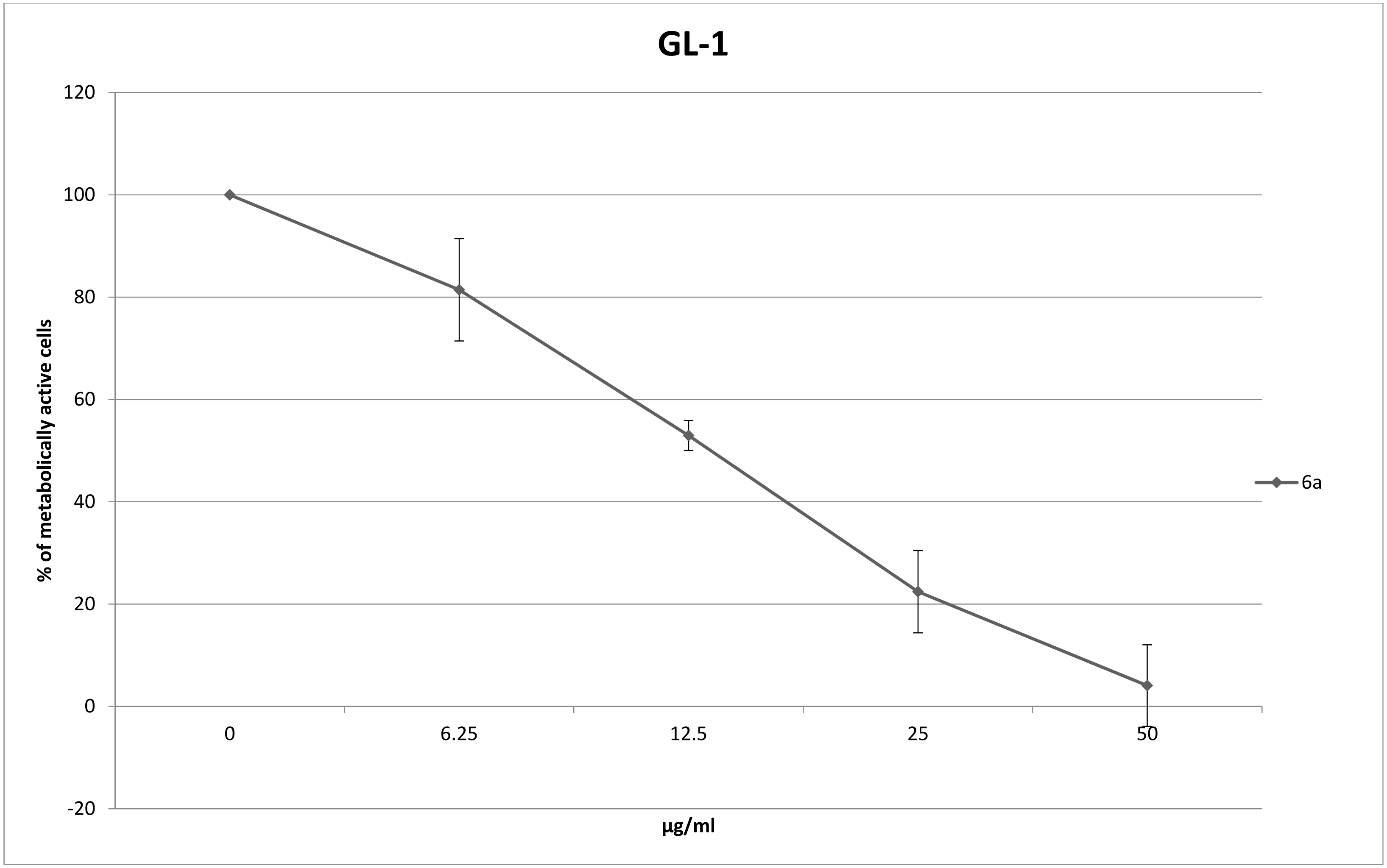

Figure S8:Dose-response curve used to calculate IC_{50} for lactone **6a** and GL-1 cell line.

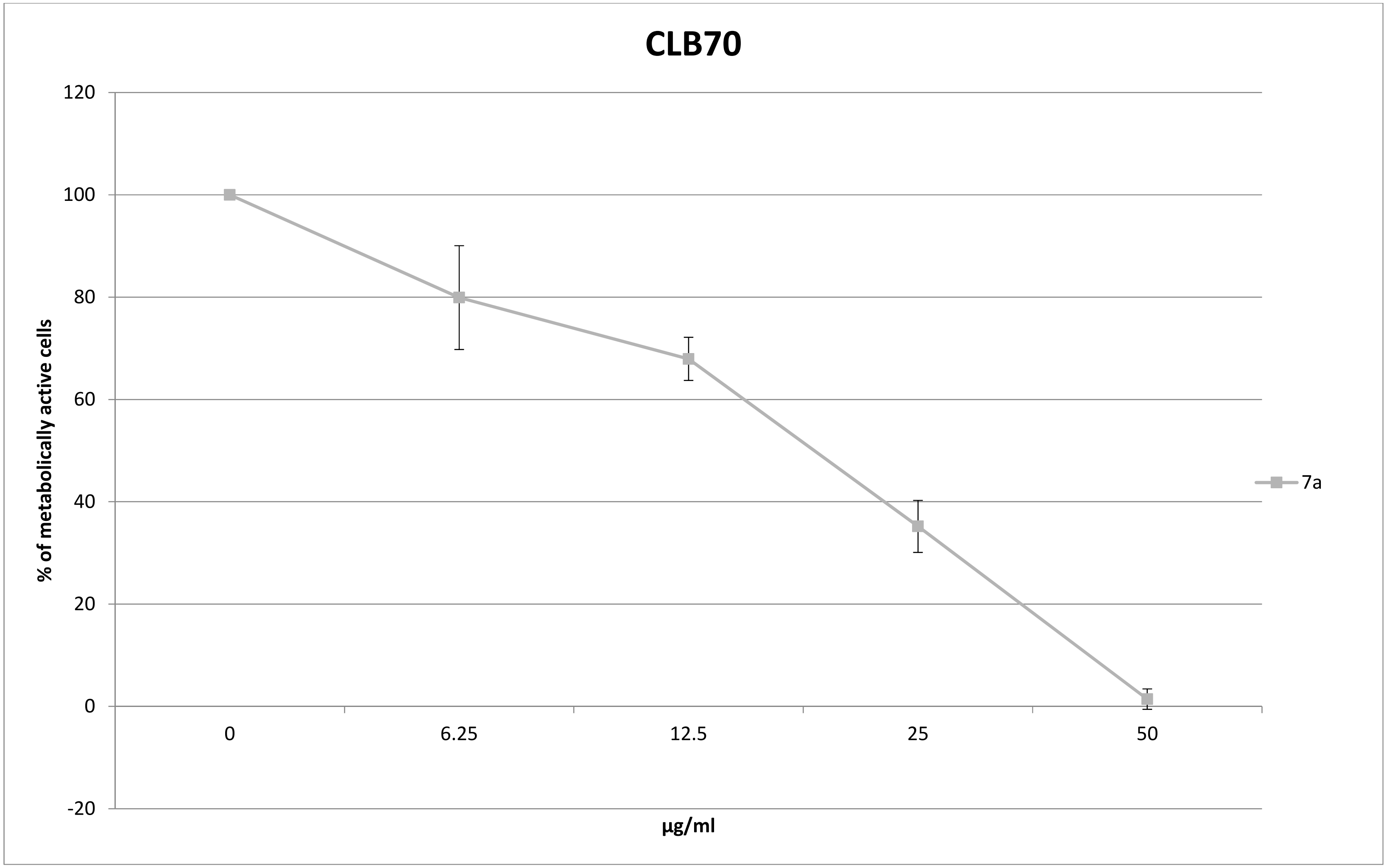

Figure S9:Dose-response curve used to calculate IC_{50} for lactone **7a** and CBL70 cell line.

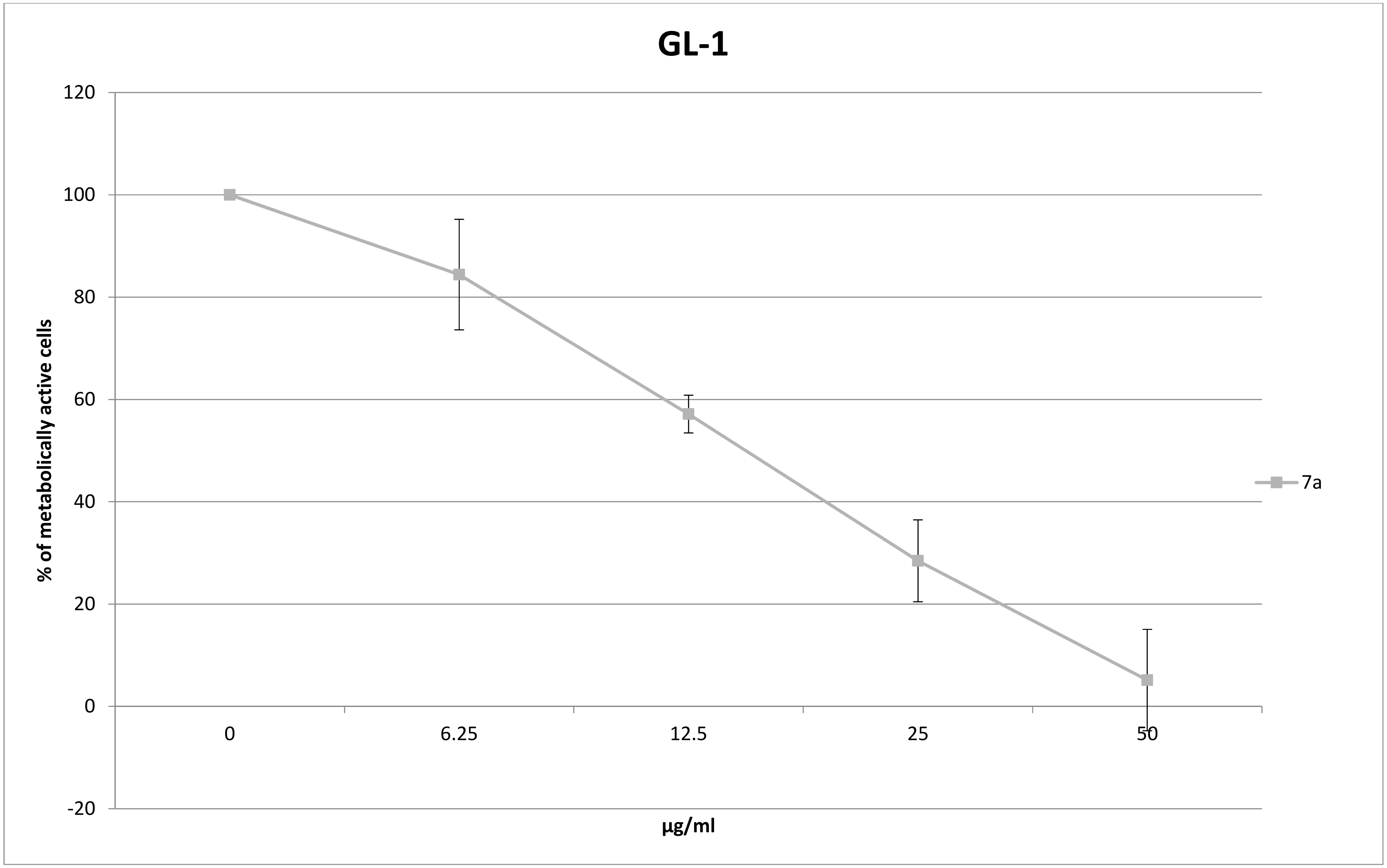

Figure S10:Dose-response curve used to calculate IC_{50} for lactone **7a** and GL-1 cell line.

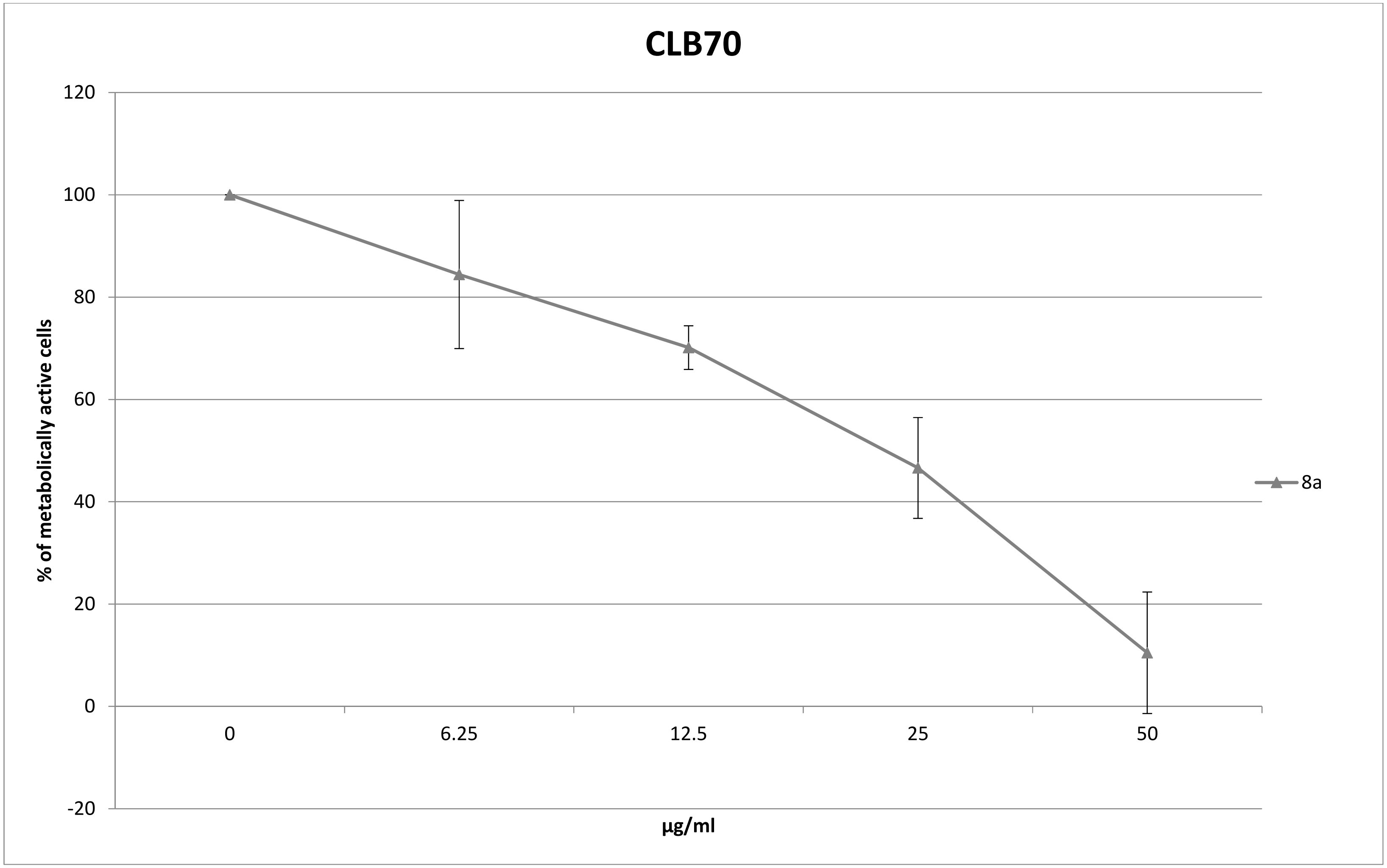

Figure S11:Dose-response curve used to calculate IC_{50} for lactone **8a** and CBL70 cell line.

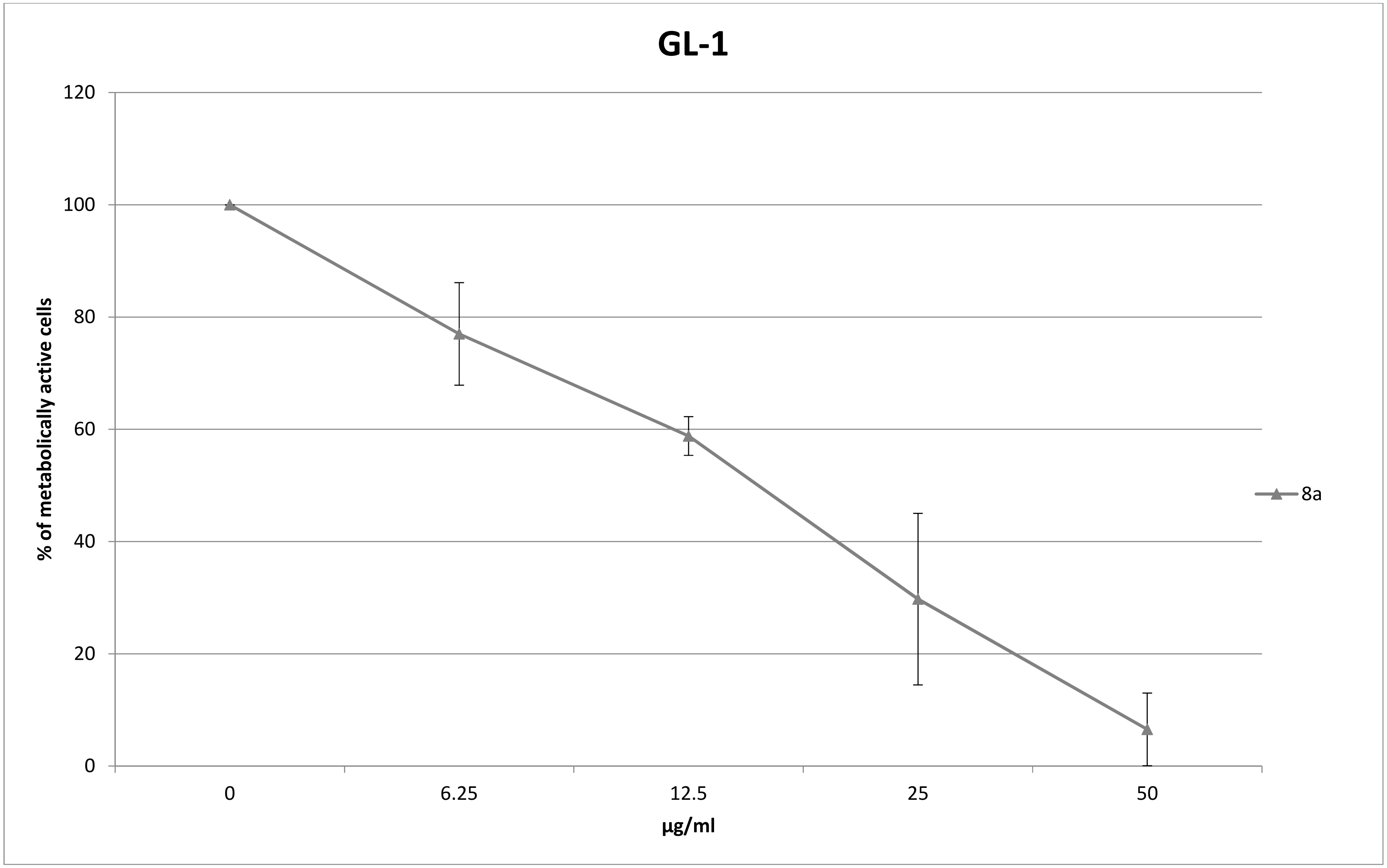

Figure S12Dose-response curve used to calculate IC_{50} for lactone **8a** and GL-1 cell line.

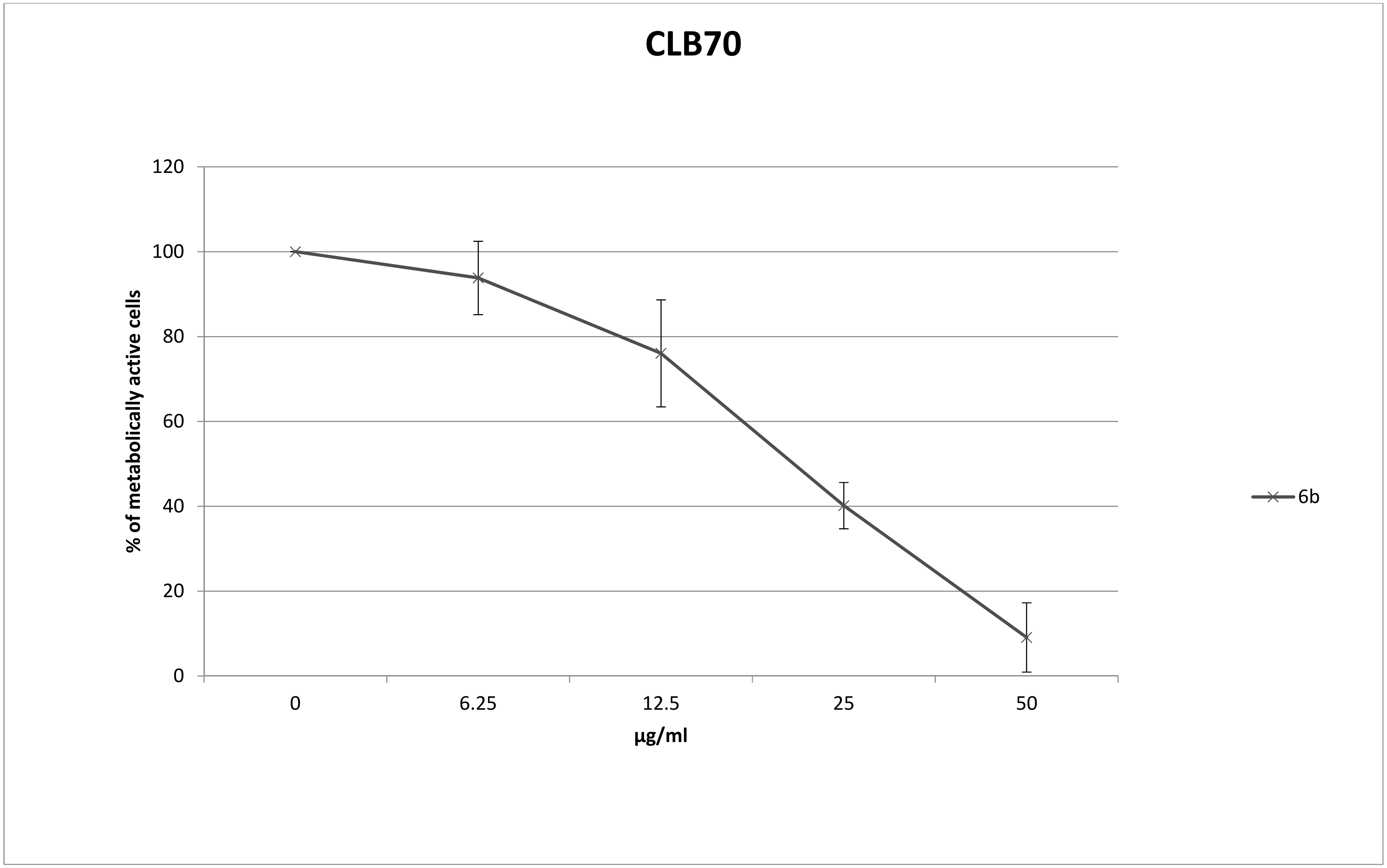

Figure S13:Dose-response curve used to calculate IC_{50} for lactone **6b** and CBL70 cell line.

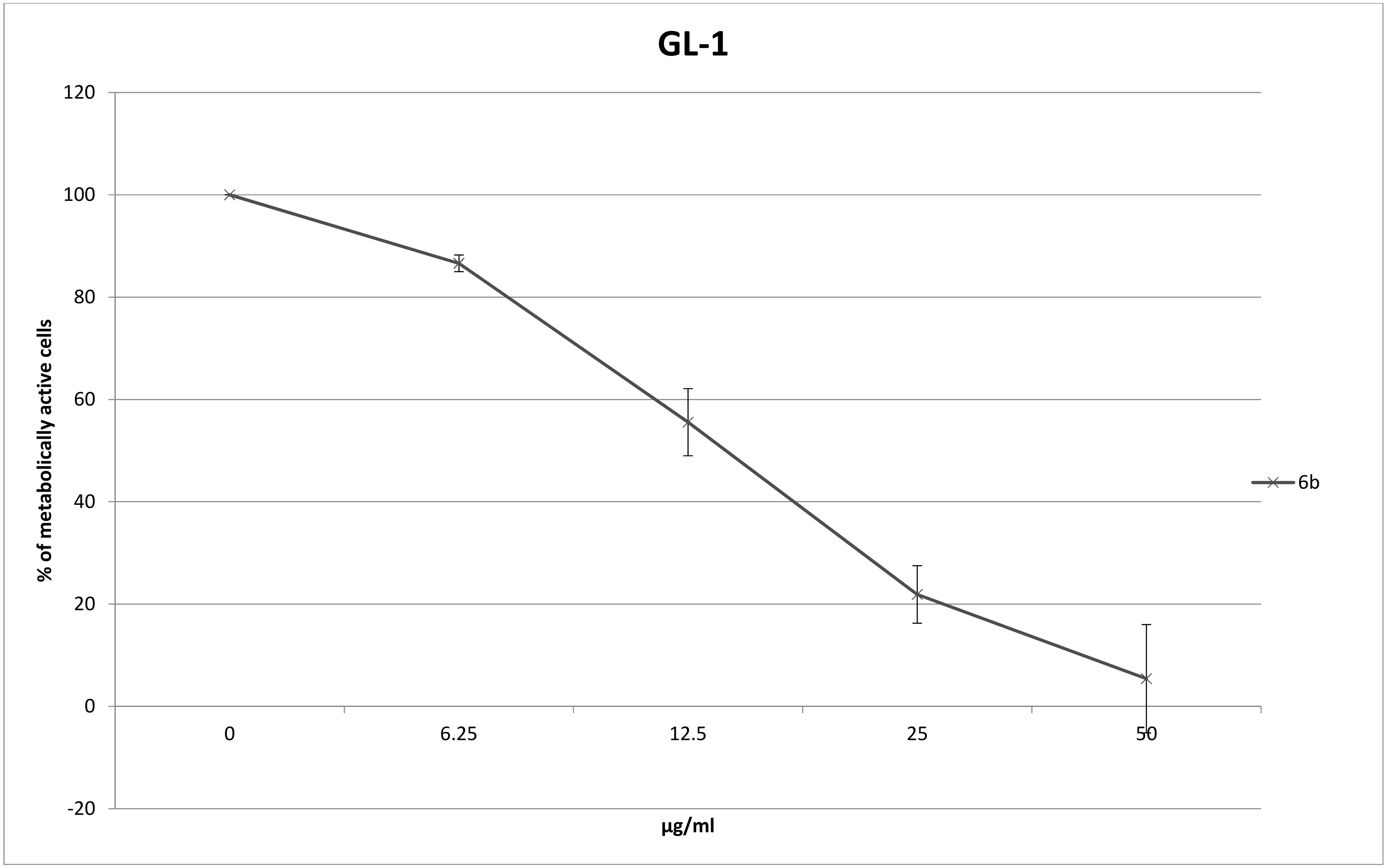

Figure S14:Dose-response curve used to calculate IC_{50} for lactone **6b** and GL-1 cell line.

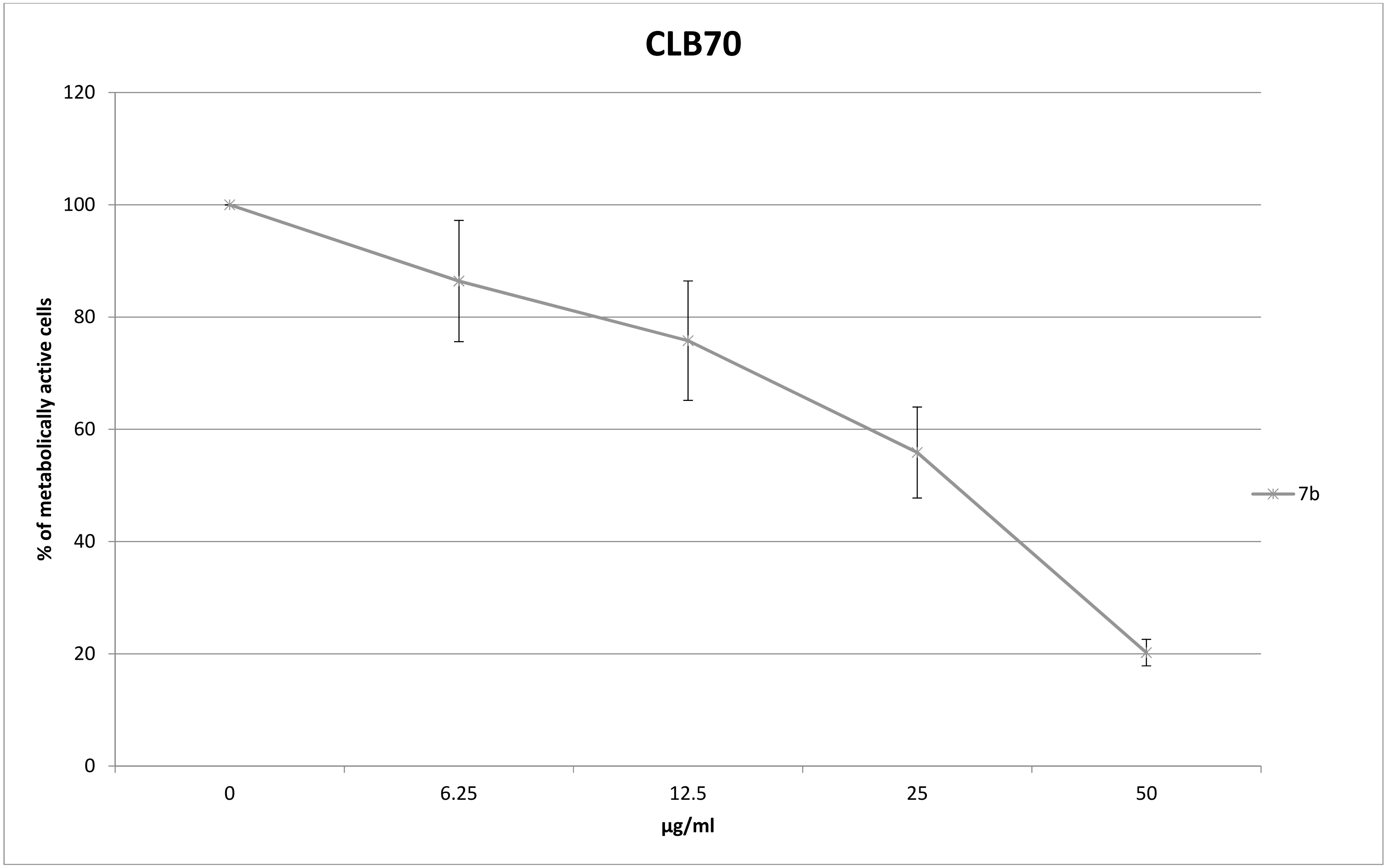

Figure S15:Dose-response curve used to calculate IC_{50} for lactone **7b** and CBL70 cell line.

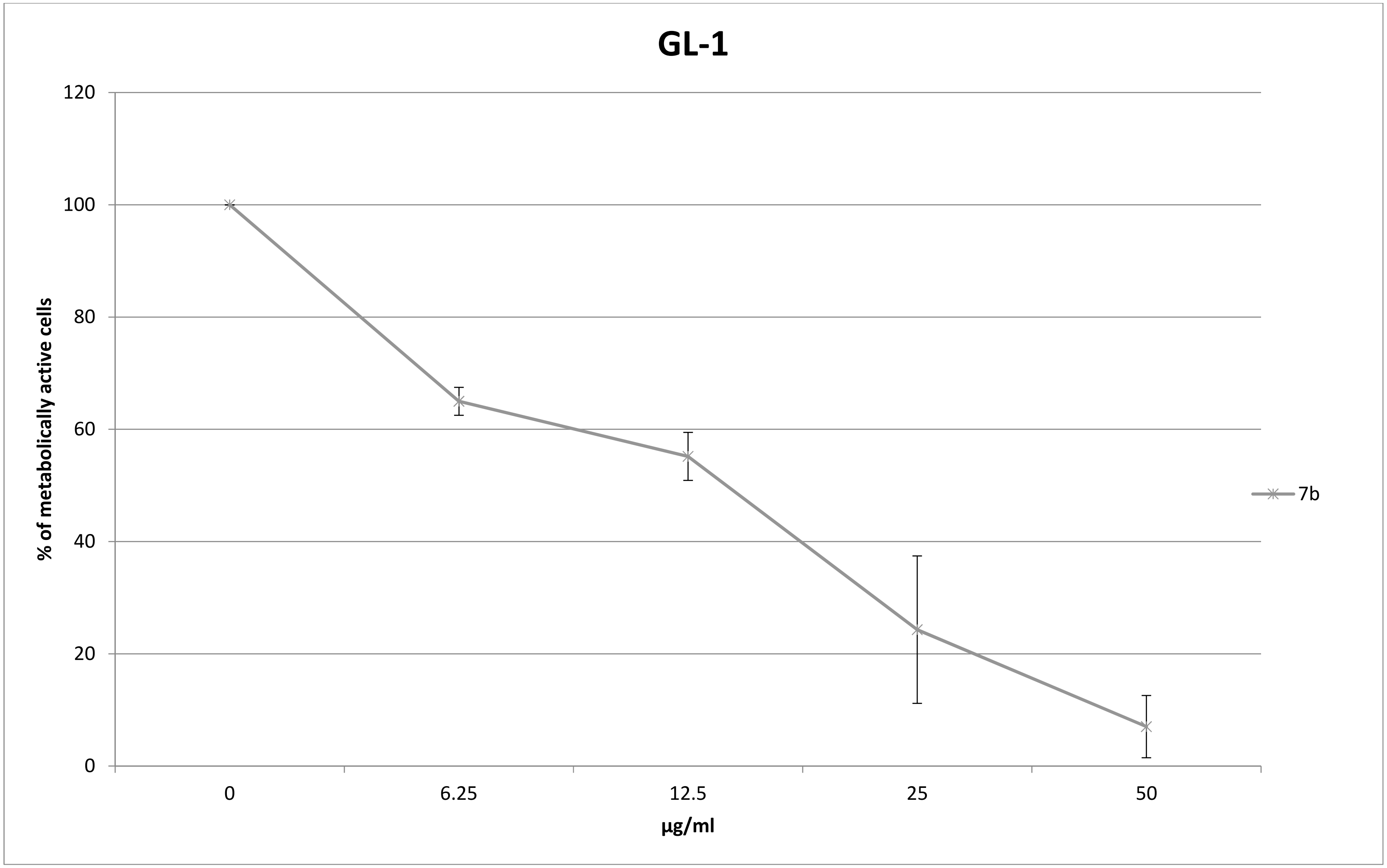

Figure S16:Dose-response curve used to calculate IC_{50} for lactone **7b** and GL-1 cell line.

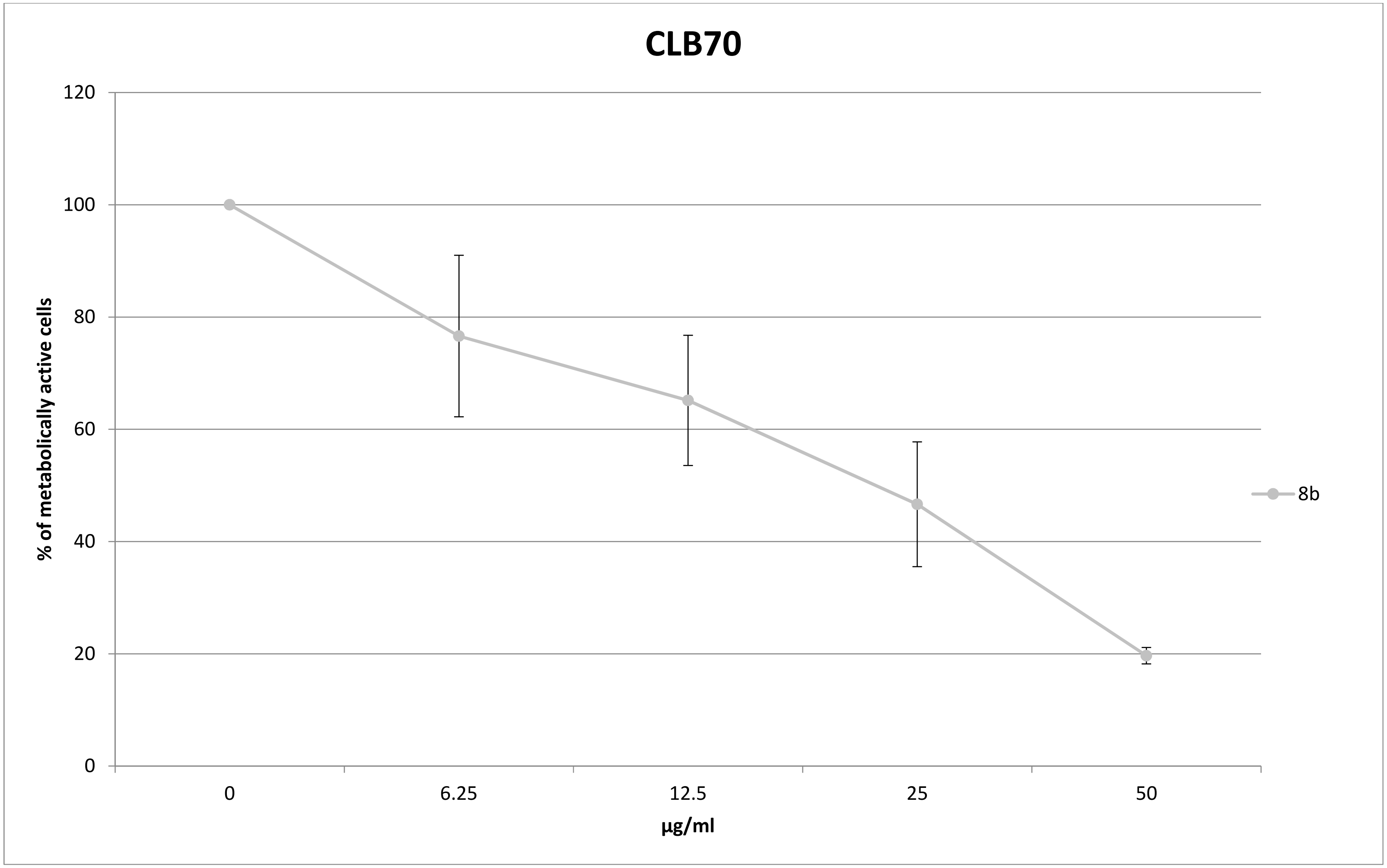

Figure S17:Dose-response curve used to calculate IC_{50} for lactone **8b** and CBL70 cell line.

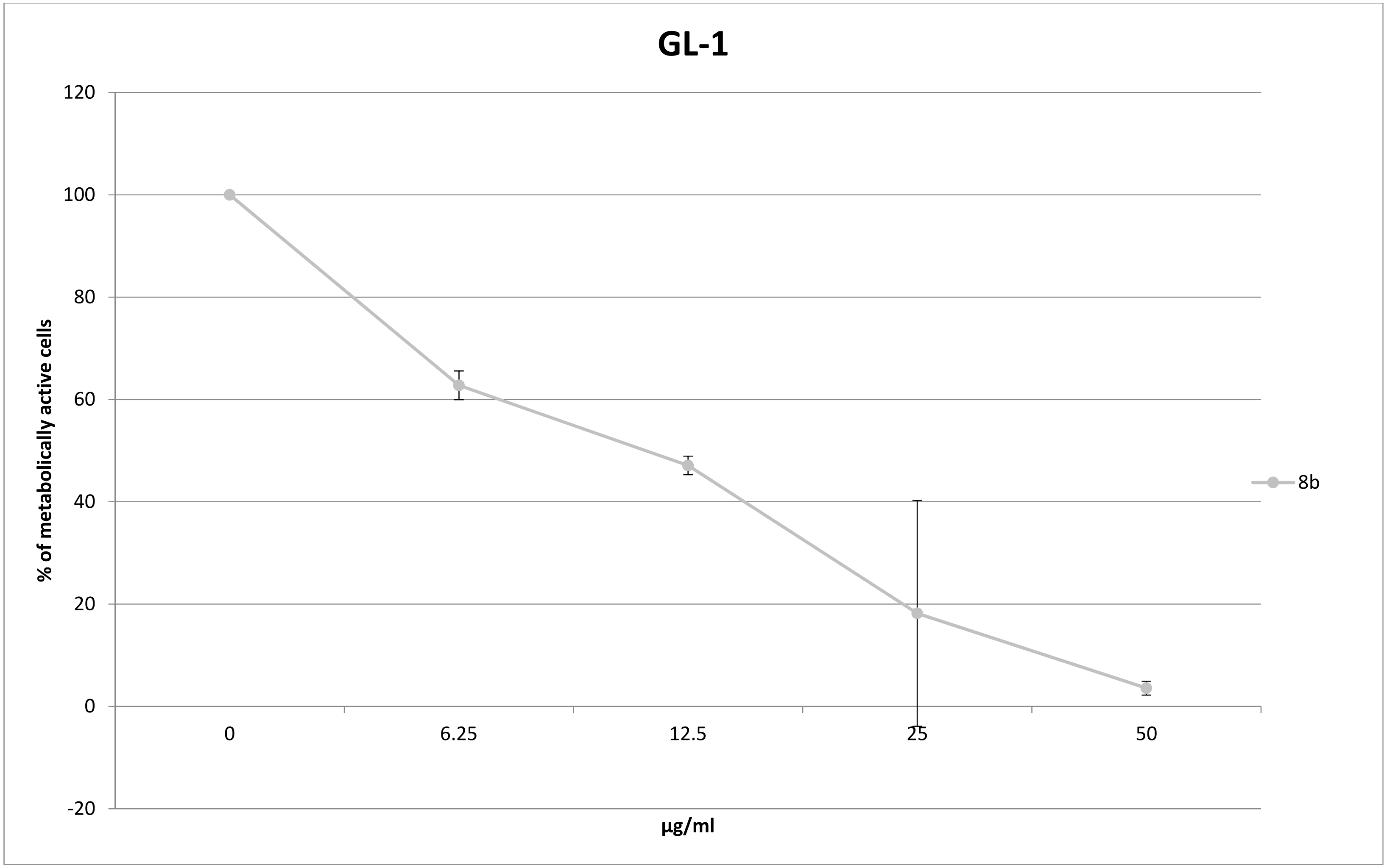

Figure S18:Dose-response curve used to calculate IC_{50} for lactone **8b** and GL-1 cell line.

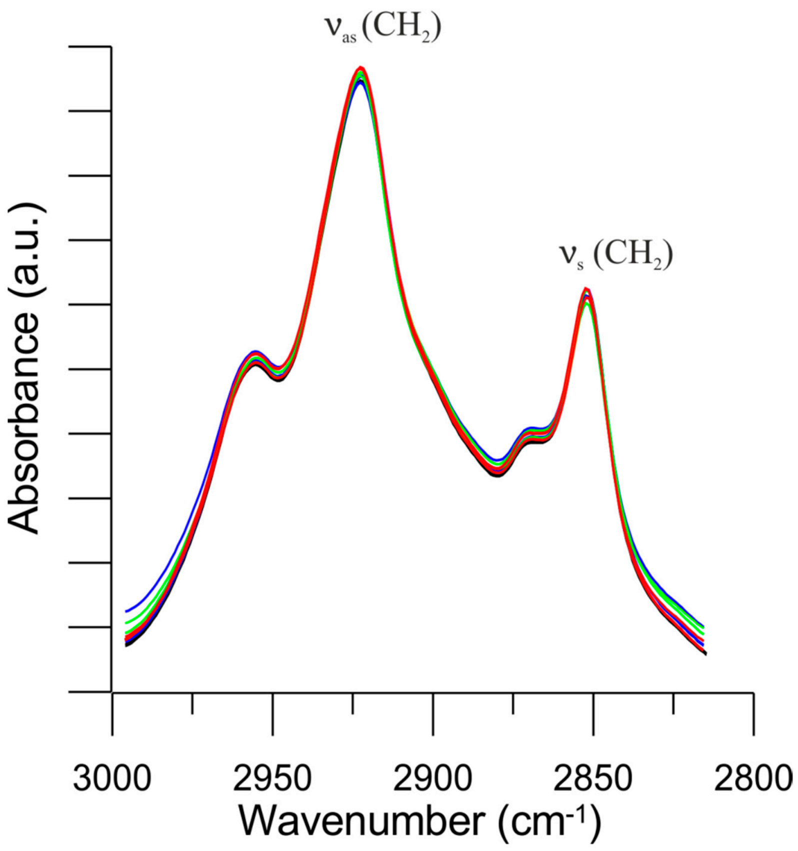

Figure S19: FT-IR spectra of erythrocyte membrane RBCMs (black lines) and of RBCMs modified with lactones (blue lines - **6a** and **6b**, green lines -**7a** and **7b**, red lines – **8a** and **8b**) for symmetric and asymmetric CH_2 stretching band.

Table S1. Hemolysis of RBCs dependent on concentration of compounds.

	**Control**	**6a**	**6b**	**7a**	**7b**	**8a**	**8b**
Concentration [mM]	Percentage of hemolysis ± SD						
**0.5**	3.530 ± 0.091	3.301 ± 0.162	3.031 ± 0.007	3.296 ± 0.085	3.256 ± 0.028	3.301 ± 0.035	3.106 ± 0.282
**1**	6.362 ± 0.542	6.197 ± 1.023	5.263 ± 0.607	7.435 ± 0.331	6.491 ± 0.409	5.218 ± 0.403	4.729 ± 0.713

Table S2. Selected bands (wave numbers cm^{-1}) of IR spectra of RBCM and RBCM+compounds (100 µM).

	**RBCM**	**6a**	**6b**	**7a**	**7b**	**8a**	**8b**
ν_s (N-C)	925.98	925.94	926.01	926.01	925.98	926.00	926.00
ν_{as} (N-C)	971.64	971.69	971.58	971.72	971.58	971.62	971.64
$\nu_s(PO_2^-)$	1064.82	1064.86	1064.77	1064.60	1064.41	1064.44	1064.42
$\nu_{as}(PO_2^-)$	1234.60	1234.26	1234.57	1234.42	1234.33	1234.35	1234.47
ν(C=O)	1739.60	1739.71	1739.94	1739.94	1739.26	1737.49	1738.11
$\nu_s(CH_2)$	2851.99	2851.90	2852.08	2852.07	2852.01	2851.93	2851.97
$\nu_{as}(CH_2)$	2922.46	2922.38	2922.66	2922.68	2922.50	2922.40	2922.53
